# Inspired by nature: Fiber networks functionalized with tannic acid and condensed tannin-rich extracts of Norway spruce bark show antimicrobial efficacy

**DOI:** 10.3389/fbioe.2023.1171908

**Published:** 2023-04-19

**Authors:** Tuula Jyske, Jaana Liimatainen, Jenni Tienaho, Hanna Brännström, Dan Aoki, Katsushi Kuroda, Dhanik Reshamwala, Susan Kunnas, Eelis Halmemies, Eiko Nakayama, Petri Kilpeläinen, Ari Ora, Janne Kaseva, Jarkko Hellström, Varpu S. Marjomäki, Maarit Karonen, Kazuhiko Fukushima

**Affiliations:** ^1^ Natural Resources Institute Finland, Latokartanonkaari 9, Helsinki, Finland; ^2^ Natural Resources Institute Finland, Teknologiakatu 7, Kokkola, Finland; ^3^ Department of Forest and Environmental Resources Sciences, Nagoya University, Nagoya, Japan; ^4^ Forestry and Forest Products Research Institute, Tsukuba, Japan; ^5^ Department of Biological and Environmental Science, Nanoscience Center, University of Jyväskylä, Jyväskylä, Finland; ^6^ Natural Resources Institute Finland, Ounasjoentie 6, Rovaniemi, Finland; ^7^ Department of Chemistry, University of Jyväskylä, Jyväskylä, Finland; ^8^ Department of Environmental Science Design, Showa Women’s University, Tokyo, Japan; ^9^ Natural Resources Institute Finland, Myllytie 1, Jokioinen, Finland; ^10^ Natural Chemistry Research Group, Department of Chemistry, University of Turku, Turku, Finland

**Keywords:** antibacterial, antiviral, bark, cellulose, phenolics, Picea abies, sidestream, tannins

## Abstract

This study demonstrated the antibacterial and antiviral potential of condensed tannins and tannic acid when incorporated into fiber networks tested for functional material purposes. Condensed tannins were extracted from industrial bark of Norway spruce by using pressurized hot water extraction (PHWE), followed by purification of extracts by using XADHP7 treatment to obtain sugar-free extract. The chemical composition of the extracts was analyzed by using HPLC, GC‒MS and UHPLC after thiolytic degradation. The test matrices, i.e., lignocellulosic handsheets, were produced and impregnated with tannin-rich extracts, and tannic acid was used as a commercial reference. The antibacterial and antiviral efficacy of the handsheets were analyzed by using bioluminescent bacterial strains (*Staphylococcus aureus* RN4220+pAT19 and *Escherichia coli* K12+pCGLS11) and Enterovirus coxsackievirus B3. Potential bonding of the tannin-rich extract and tannic acid within the fiber matrices was studied by using FTIR-ATR spectroscopy. The deposition characteristics (distribution and accumulation patterns) of tannin compounds and extracts within fiber networks were measured and visualized by direct chemical mapping using time-of-flight secondary ion mass spectrometry (ToF-SIMS) and digital microscopy. Our results demonstrated for the first time, how tannin-rich extracts obtained from spruce bark side streams with green chemistry possess antiviral and antibacterial properties when immobilized into fiber matrices to create substitutes for plastic hygienic products, personal protection materials such as surgical face masks, or food packaging materials to prolong the shelf life of foodstuffs and prevent the spread of infections. However, more research is needed to further develop this proof-of-concept to ensure stable chemical bonding in product prototypes with specific chemistry.

## Introduction

Infections are caused when microorganisms, such as bacteria, fungi or viruses, enter the human body and cause damage. These pathogenic microorganisms can spread in various ways, such as skin contact or inhalation of airborne droplets. Infectious diseases are the leading cause of morbidity and mortality worldwide (Bachir raho and Abouni, 2015). Viruses cause epidemics on a yearly basis, many of them leading to hospitalizations and even deaths, and even pandemics such as the present COVID-19 pandemic caused by SARS-CoV-2. Effective preventive measures and biocidal products are thus needed to combat pathogens. In addition to airborne droplets of infective microbes, contaminated material surfaces are also an important route of transmission. Thus, materials with functional antimicrobial activities that are safe, non-toxic, and preferably more effective than those currently on the market are desperately needed.

At the same time, there is a global need to reduce plastic waste by creating biobased and biodegradable materials. In 2015, the demand for plastics in Europe reached 46 million tons. Millions of tons of plastic litter in the oceans have alarmed the public, emphasizing the urgent need to innovate alternative materials offering functionalities similar to those of plastics. Bioeconomy strategies in the EU and the European-wide strategy on plastics in the circular economy highlight the importance of replacing fossil-based raw materials with biobased ones, using materials and side-products efficiently, and creating new smarter products that are renewable, recyclable, and provide higher added value (European Commission, 2018).

**FIGURE 1:**
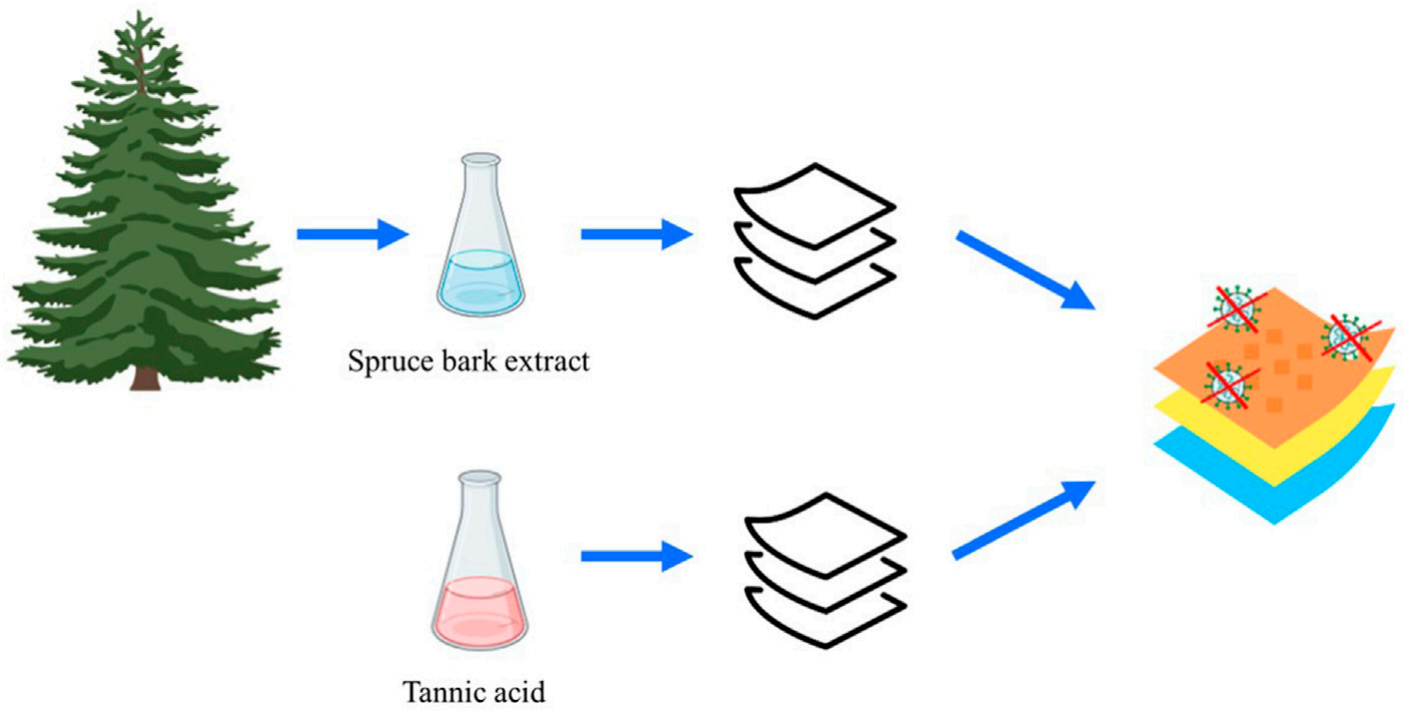
Schematic approach of the study to produce bioinspired functional materials with spruce bark extract and tannic acid.

Biomimicry is an approach to innovate novel sustainable solutions—products, materials, ways-of-manufacturing—inspired by nature. The new innovations rise when biology and technology meet. Nature-inspired inventions are needed to transform societies from fossil-based into carbon-neutral ones. Forest trees accumulate chemical compounds with antimicrobial, antioxidant, and hydrophobic properties that are often linked to monomeric and polymeric condensed polyphenolic compounds, such as tannins. We have revealed how naturally bioactive phenolic compounds accumulate within bark tissues and cells as a sophisticated chemical defense barrier (Jyske et al., 2015; Jyske et al., 2016; Jyske et al., 2020a). In the context of biomimicry, bark can serve as a biological model to create functional multilayer surfaces of fiber products (Figure 1). In Finland alone, over one million tons of Norway spruce (*Picea abies* (L.) Karst.) bark is annually produced in round wood processing, providing a potential source for high-value extracts (Statistics Finland OSF, 2021). Naturally, bioactive biopolymers with antibacterial and antiviral properties (Välimaa et al., 2020; Reshamwala et al., 2021; Tienaho et al., 2021) can be obtained from bark by applying extraction and fractionation processes of green chemistry (Kilpeläinen et al., 2014; Rasi et al., 2019). The isolated biopolymers, fibers, and extracts can be used as such or further chemically modified and used as building blocks for functionalized consumer products, such as hygienic products, protective equipment, and packaging, or even in medical care equipment (Baranwal et al., 2022).

Personal protection and hygienic products as well as packaging materials, especially food contact materials, are regulated in the EU in the REACH legislation to ensure consumers’ safety (i.e., the Regulation for Registration, Evaluation, Authorization and Restriction of Chemicals; European Chemical Agency) (European Chemical Agency, 2023). The positive list indicates materials already accepted, and clear guidance is given to seek approval of new chemicals or products for skin and food contact materials. Regulations can be used as a guide during research and development, and relevant data needed for approval of applications can be collected. For instance, the production of antimicrobial personal protection equipment (such as surgical face masks) or food packaging requires that the active components do not deteriorate user health or migrate to foods (European Commission, 2021).

The aim of this research was to study the potential of naturally bioactive, tannin-rich extracts of Norway spruce bark to create smart functional fiber surfaces. Tannic acid was used as a commercial reference with known antiviral efficacies (Kaczmarek, 2020). Bark extract was obtained from industrial bark side streams. Research focused on **1)** production of extract with hot water in pilot scale, **2)** reveal extract chemical composition and **3)** bioactivity, and **4)** analyze fiber binding potential and thus usability in biobased fiber applications, mimicking the natural cellular defense barrier of bark. Test matrices of fiber-networks were prepared to study different approaches in incorporating the functionality into fiber networks and to test their functionality (i.e., antibacterial, and antiviral surface properties) aiming at further development of smart lignocellulosic fiber products for personal protection or other functional use. We hypothesized that **1)** tannic acid and tannin-rich bark extract show broad spectrum antimicrobial activity against both bacteria and viruses; **2)** tannic acid and tannin-rich extract as immobilized into fiber samples maintain their antimicrobial activity; **3)** tannic acid and tannin-rich extract are evenly localized in fiber matrices, and **4)** surface techniques would reveal whether the interaction between bioactive compounds and fiber surface molecules are reversible with non-covalent forces or irreversible via formation via covalent bonds. Additionally, novel techniques were used, including bioluminescent imaging of bacterial plates, antiviral efficacy tests by enterovirus, morphological analysis by laser microscopy, chemical mapping of bioactive compounds on sample surfaces by ToF-SIMS, and semiquantitative analysis of potential chemical bonds between impregnated bioactive compounds and the target fiber surfaces by FTIR-ATR spectroscopy, supported by the chemical characterization of the extract by GC‒MS and LC‒MS, and chemical quantitation of extractives by GC, stilbenes by HPLC and condensed tannins by UHPLC after their thiolytic degradation.

## Materials and methods

### Bark material sourcing

Industrial Norway spruce (*P. abies* (L.) Karts.) bark from a sawmill was used for pilot-scale extraction. Bark originated from a sawmill located in Haapajärvi (63°7′N, 25°3′E) in Finland, and it was retrieved on 4 September 2019, immediately after debarking of trees and industrial shredding of bark. Spruce bark was further comminuted with a shredder having a screen of 25 mm × 25 mm mesh (Eliet Super Prof 2000; LECTURA GmbH, Nuremberg, Germany). The bark was stored at +5°C until extraction, which was carried out on 12 September 2019.

### Extraction

Pilot-scale extraction of spruce bark was carried out with a 300-L reactor for 40 min at 88°C in batch mode at normal atmospheric pressure (Kilpeläinen et al., 2014). A total of 89 kg of fresh bark, which equals 38 kg of dry bark, was added into the reactor. A total extract of 211 L was collected into a 1000-L container. Samples from the crude extract were taken and stored in a freezer at −20°C prior to further chemical analyses. Hot water extract was purified using 7 kg of XAD7HP adsorbent (Sigma‒Aldrich, Saint-Louis, MO, United States), which was packed in a 62 cm glass column. Approximately 53 L of extract was eluted through a column, the column was washed with water, and polyphenols were eluted out of the adsorbent using 95% ethanol. The eluted purified extract was first evaporated to a small volume with a rotary evaporator, after which the concentrated extract was freeze-dried. A freeze-dried, tannin-rich product of hot water extraction (TW8.1, hereafter referred to as TW) was used for handsheet studies.

### Chemical characterization of the extract

For the analysis of individual compounds in the extract, 2–3 mg of extract (as dry matter) and, with quantitative analysis, 100 μg of internal standards (heneicosanic acid and betulinol) were derivatized after drying with a nitrogen stream for GC with a mixture of BSTFA and TMCS (99:1, v/v, respectively) (Halmemies et al., 2021). Derivatization of samples was performed by keeping them at 70°C for 1 hour. Three replicate samples were prepared for quantitative analyses. Quantitative analysis of the individual compounds was carried out on an Agilent 6850 Series GC System with an HP-5 (30 m × 0.32 mm, film thickness 0.25 μm) column. The injector temperature was 290°C, the injection volume was 1 μL, the injection mode was pulsed splitless, and the detector temperature was 300°C. The GC oven temperature program was 1.5 min at 100°C, followed by an increase of 6°C/min to 180°C, 10 min at 180°C, an increase of 4°C/min to 290°C, 13 min at 290°C, an increase of 4°C/min to 300°C and 20 min at 300°C. Qualitative analysis of the individual components was carried out with an Agilent 6890 Series GC System equipped with a 7,683 injector and an Agilent HP 5973 mass selective detector (MSD). The MS full scan mode was used, and the mass range was from 50 m*/z* to 550 m*/z*. The capillary column used was the same, and the GC oven temperature program was the same as that used in the quantitative analyses.

In addition to GC analyses, stilbenes were analyzed with HPLC by the method developed by Halmemies et al. (Halmemies et al., 2021), see also Jyske et al. (Jyske et al., 2020b; Jyske et al., 2022) The amount and composition of sugars and hemicelluloses in the extract were determined with acidic methanolysis (Sundberg et al., 1996) with the setup described in the earlier study (Kilpeläinen et al., 2014). Extracts were treated for 3 h. Resorcinol was used as an internal standard. Silylated samples were analyzed with a Shimadzu GC-2010 gas chromatograph (Kyoto, Japan) equipped with an HP-1 column (25 m length, 0.2 mm ID and 0.11 μm film thickness) and a flame ionization detector (FID).

Condensed tannins (CTs, proanthocyanidins) were determined by ultrahigh-performance liquid chromatography (UHPLC) after thiolytic degradation according to previous study (Korkalo et al., 2020). Briefly, samples were weighed (20–30 mg) into 1.5 mL Eppendorf vials, and 1 mL of depolymerization reagent (3 g cysteamine/4 mL 13 M HCl/56 mL methanol) was added. The vials were sealed and incubated for 60 min at 65°C, after which the degradation products, i.e., free flavan-3-ols (terminal units) and their cysteaminyl derivatives (extension units), were determined by UHPLC equipped with diode array detection (DAD) and fluorescence detection (FLD).

### Chemical characterization of tannic acid

Commercial tannic acids are mixtures of tens of polyphenols, and the polyphenol contents differ within tannic acids (Salminen and Karonen, 2011). Therefore, the tannic acid used (Sigma‒Aldrich, Saint-Louis, MO, USA) was carefully analyzed by UHPLC‒MS/MS according to earlier study (Karonen et al., 2021). The chemical composition of this reference material is presented in Supplementary Figure S5.

### Antiviral efficacy of tannin-rich bark extract (TW) and commercial tannic acid (TA)

The antiviral activity of tannin-rich bark extract (TW) and commercial tannic acid (TA) against enterovirus coxsackievirus B3 (CVB3; Nancy strain) was performed using a cytopathic effect (CPE) inhibition assay, modified from earlier study (Reshamwala et al., 2021). The virus was obtained from American Type Culture Collection (ATCC, Manassas, VA, USA). Briefly, A549 cells (ATCC) at a density of 12,000 cells/well were cultured in 96-well flat-bottomed microtiter plates (Sarstedt, Numbrecht, Germany) for 24 h at 37°C using Dulbecco’s Modified Eagle Medium (DMEM; Gibco, Paisley, United Kingdom) supplemented with 10% fetal bovine serum (FBS; Gibco, Paisley, United Kingdom), 1% GlutaMAX (Gibco, Paisley, United Kingdom) and 1% penicillin/streptomycin antibiotics (Gibco, Paisley, United Kingdom). The next day, the virus was pretreated with different concentrations of TW or TA for 1 h at 37°C. The virus titre in the virus-compound mix was 2 × 10^7^ PFU/mL. Following incubation, the virus-compound mix was further diluted 10-fold using DMEM to achieve an MOI of 10. Then, the mix was added to the cells for incubation for 24 h at 37°C. A mock infection without the compound and virus was used as a control, and a virus control without the compound was also used as another control during the assay. After 24 h, the cells were washed with phosphate-buffered saline (PBS) and then fixed and stained using CPE dye (0.03% crystal violet, 2% ethanol, and 36.5% formaldehyde) for 10 min. Following the water washes, the viable cells were lysed using lysis buffer (0.8979 g of sodium citrate and 1 N HCl in 47.5% ethanol). The absorbance was measured spectrophotometrically at 570 nm using a VICTOR™ X4 (PerkinElmer, Turku, Finland) multilabel reader.

### Antibacterial efficacy of tannin-rich bark extract (TW) and commercial tannic acid (TA)

Tests were conducted according to the procedure described earlier (Tienaho et al., 2021). In brief, the constitutively luminescent light signal emitting bacterial biosensor strains *E. coli* K12+pcGLS11 and *S. aureus* RN4220+pAT19 were used. The undisturbed luminescent light production produced during the normal metabolism of the strains was 10^9^ photons/s/cm^2^/sr (sr = steradian, unit of solid angle) for the *E. coli* strain (4.2 × 10^8^ cfu/mL) and 10^6^ photons/s/cm^2^/sr for the *S. aureus* strain (4.3 × 10^9^ cfu/mL). The strains indicate the presence of antibacterial substances by a measurable decrease in the produced luminescent light signal. Strains were stored at −80°C and cultivated for approximately 16 h at 30°C (*E. coli*) and 37°C (*S. aureus*) on lysogeny agar plates (LA) (tryptone 10 g/L; yeast extract 5 g/L; NaCl 10 g/L; and agar 15 g/L). The *E. coli* plates were supplemented with 10% (v/v) sterile filtered phosphate buffer (PB) (1 M, pH 7.0) and 100 μg/mL ampicillin and *S. aureus* plates with 5 μg/mL erythromycin. Bacterial stocks were prepared by inoculating a single colony in lysogeny broth with the same supplementations as LA plates. Stocks were again cultivated for approximately 16 h at 300 rpm shaking at 30°C (*E. coli*) and 37°C (*S. aureus*). The used samples TW (TW) and tannic acid (TA) were diluted in double-distilled water to achieve concentrations of 2.50, 1.25, 0.625, 0.313, 0.156, 0.078, and 0.039 mg/mL per microplate well. The controls used were an ethanol concentration of 18% per microplate well (positive control) and double-distilled water (negative control). Samples and controls (50 µL) were pipetted in triplicate into opaque white polystyrene microplates, and a constant volume of 50 µL of bacterial inoculations was added to all microplate wells. The luminescence was then immediately measured using a Varioskan Flash Multilabel device (Thermo Scientific, Waltham, Massachusetts, United States) once every 5 min for 95 min at room temperature, and the plate was briefly shaken before every measurement. The results are expressed as inhibition percentages (inhibition%) at 50 min of measurement. Error bars represent the standard deviations between the sample triplicates.

### Handsheets preparation

#### Handsheet preparation for the experiments

Handsheets were prepared at the Tampere University of Applied Sciences from unbleached thermomechanical pulp of spruce. The pulp was produced by UPM Biofore, the consistency of the pulp was 36 g/L, and the freeness (CSF) was 60 mL. Sheets were prepared according to the ISO 5269 standard. The sheets produced had square masses of 60 g/m^2^ and 130 g/m^2^ with densities of 305 kg/m^3^ and 340 kg/m^3^, respectively.

#### Handsheet impregnation by dipping with extract (TW) and tannic acid (TA)

Pieces of handsheets were incubated in sodium acetate buffer containing TW bark extract (TW) or tannic acid (TA). Incubation was performed in Petri dishes (3 cm diameter) by immerging handsheet strips (20 mm × 25 mm) for 1 hour in sodium acetate buffer (1 mL, 25 mM, pH 4.5) with Tween 40 (0.1% (w/v)) in the dark. After an hour, the strip was temporarily lifted from the Petri dish, and 1 mL of TW solution (4% or 8% w/v in acetate buffer, 25 mM) or 1 mL of tannic acid solution (84 mg or 168 mg in 25 mL of acetate buffer, 25 mM, corresponding to the quantity of condensed tannins in TW solutions) was added to the dish and mixed. The strip was laid back into the dish, and incubation was continued for 24 h at 25°C in the dark. Control samples were treated under similar conditions without TW or TA. The handsheet strips were steeped twice in 150 mL of distilled water for 30 min, after which strips were washed two times extensively in distilled water for 1 h with 30 mL of water and finally air-dried in an oven at 25°C in the dark overnight. Six replicates were prepared from each test sheet. The specimen types for further analysis were untreated blank and handsheets with 2% TA, 4% TA, 2% TW, and 4% TW (i.e., TA2%, TA4%, TW2%, and TW4%, respectively).

### Antiviral efficacy assays of tannin-enriched handsheets

Enterovirus CVB3 was used to assess the antiviral efficacy of the paper samples. For antiviral testing, A549 cells at a density of 12,000 cells/well were cultured in 100 µL of DMEM supplemented with 10% FBS, 1% GlutaMAX and 1% penicillin/streptomycin antibiotics on a 96-well flat-bottomed microtiter plate for 24 h in a humidified 5% CO_2_ incubator at 37°C. The following day, the paper samples were cut into 1 cm^2^ pieces and placed inside a 12-well plate at room temperature and in humid conditions (over 90% relative humidity). Ten microliters of CVB3 (2 × 10^5^ PFU/mL) was placed on the surface of each of the 1 cm^2^ paper samples for 5 min. After incubation, 990 µL of 10% DMEM was added to the paper sample, and then a 12-well plate was placed on a rocker for 15 min to detach the virus. Next, the medium was collected, and samples were added to the A549 cells (MOI - 0.1) in 96-well plates and incubated for 48 h or until a CPE was observed. A virus without the paper samples was used as a positive control, and a mock infection without the virus and paper samples was used as a negative control for the experiment. Paper samples without the virus were also tested as described above to assess their cytotoxicity on A549 cells. After 48 h, the cells were washed twice with phosphate-buffered saline (PBS) solution and then stained with CPE stain (0.03% crystal violet, 2% ethanol, and 36.5% formaldehyde) for 10 min at room temperature. Following two washes with water to remove the excess stain, cells were lysed using lysis buffer (0.8979 g of sodium citrate and 1 N HCl in 47.5% ethanol). Finally, the viable cells in the wells were determined by measuring their absorbance spectrophotometrically at 570 nm using the PerkinElmer VICTOR™ X4 multilabel reader. This assay was performed twice independently.

### Bacterial imaging tests for tannin-enriched handsheets on agar plates

Same constitutively luminescent light signal emitting bacterial biosensor strains *E. coli* K12+pcGLS11 and *S. aureus* RN4220+pAT19, as described above, were used in the assay. An aliquot of 7 mL of bacterial inocula grown overnight was added for every 100 mL of soft agar (LA but with 7.5 g/L (50%) agar content) supplemented with 10% (v/v) sterile filtered phosphate buffer (PB) (1 M, pH 7.0), 100 μg/mL ampicillin (*E. coli*) and 5 μg/mL erythromycin (*S. aureus*) at approximately 50°C. The soft agar containing bacteria was mixed gently and rapidly poured over Petri plate triplicates containing approximately 1 cm × 1 cm pieces of the test sample sheets on a thin layer of LA before the soft agar started to solidify. The plates were then inoculated at 30°C (*E. coli*) or 37°C (*S. aureus*) overnight (approximately 16 h) and finally scanned using a SPECTRAL Lago X *in vivo* imaging system (Spectral Instruments Imaging, AZ, United States) in luminescence and image overlay mode. The exposure time was set at one second, small to medium binning (2×–4×) was used, the field of view (FOV) was 25 × 25 cm (for 4 Petri plates at once), and the object height was 1.5 cm. Data were then handled using Aura Spectral Instruments Imaging Software version 3.2. Circular regions of interest (ROIs) were chosen above and around the test sample sheets, and the results were obtained in Photons/s/cm^2^/sr. To change the results into more comparable units between bacterial inoculations, the sample result averages from the three plate replicates were divided with control plate (containing only bacterial inoculation soft agar and no sample) ones to obtain units of impact factor (IF) and expressed in inhibition percentages (inhibition%) (Tienaho et al., 2015). The error bars represent the coefficient of variation between the sample sheets on three plate replicates.

### Surface properties and topochemistry

#### FTIR-ATR spectroscopy

To examine the TA and TW extracts and surfaces of the untreated handsheets (control samples) and sheets treated with the extract (20 mm × 25 mm), the samples were analyzed by FTIR with the single reflection ATR technique (attenuated total reflectance, ZnSe crystal plate). The ATR analyses were executed using a Shimadzu IR Prestige-21 spectrometer (Ordior Oy, Helsinki, Finland) equipped with a DLATGS detector and Shimadzu IRsolution 1.40 2007 software. Spectra were acquired in absorbance mode using 60 scans at a resolution of 4 cm^-1^ in the range of 4,000–400 cm^-1^. All samples were randomly sampled and analyzed with three parallel measurements for repeatability and consistent analysis.

#### ToF-SIMS analysis for the chemical mapping of handsheet surfaces

First, we analyzed the candidate peaks of the TA and TW extracts. Then, we analyzed the localization and accumulation of the selected candidate peaks within the paper samples by using the methodology shown earlier (Kuroda et al., 2014; Jyske et al., 2016). Briefly, paper samples were cut into smaller pieces (ca. 15 × 15 mm) and attached to the sample holder of the ToF-SIMS system. ToF-SIMS measurements were carried out using a TRIFT III spectrometer (ULVAC-PHI, Kanagawa, Japan). The pressure in the ToF-SIMS specimen chamber was ca. 1 × 10^−9^ Pa. Positive and negative spectra were obtained by using 22 keV Au_1_^+^ ions at a current of 5–7 nA. The measured surface areas were 200 × 200 μm^2^ and 500 × 500 μm^2^, with a sample surface primary ion dosage of <2.3 × 10^9^ and secondary ion counts of >2 × 10^6^. Each image (raw data) contained a full mass spectrum with a resolution of 256 × 256 pixels. The mass spectrum data for the estimated candidate peaks of the TA and TW extracts within the paper samples were analyzed as spectral data and images.

The compounds within the tannic acid-treated paper samples were identified by the direct comparison of their peaks at the corresponding mass-to-charge ratio (*m*/*z*) with the peaks of initial tannic acid components, as analyzed by ToF-SIMS (Supplementary Figure S1; Supplementary Tables S1, S2). The freeze-dried TW extract was also analyzed to identify candidate peaks (Supplementary Figure S2; Supplementary Tables S1, S2). The standard spectra of the initial components of tannic acid and extract under dried conditions were measured by ToF-SIMS (both spectral and image-mode data). The data were then used for the analysis of the localization and accumulation of the tannic acid and extract within paper samples. For the spatial analysis of the compound distribution and accumulation within handsheets, the corresponding peak intensities at the estimated *m*/*z* were detected and normalized to the total secondary ion counts (i.e., relative peak intensities) in each image.

#### Laser microscopy for roughness analysis of handsheet surfaces

Laser scanning confocal microscopy was used to visualize surface texture and quantitatively determine surface thickness and the three-dimensional (3D) surface roughness of the produced fiber matrices finished with tannic acid and tannin-rich extract TW. A Keyence VK-X laser microscope (Itasca, Illinois, United States) and the accompanying multifile analysis application were used for the measurements. The laser microscope enables non-contact observation of surface properties, and as the light source with a short wavelength and high linearity, it is also effective for samples with low surface reflectance and white color samples, which are difficult to observe with visible light. Sa indicates the arithmetical mean height (average of the absolute values of the heights of the points in the defined area). This parameter is the areal (three-dimensional) expression of Ra: arithmetic mean roughness, which is generally used to evaluate surface roughness (ISO 4287:1997). The sample surface was photographed at ×5 magnification using a laser and visible light at a minimum of five randomly selected points. The measurement method complies with the international standard ISO 25178 Surface properties (measurement of surface roughness). The thickness of the sample paper was determined from the difference in position between the mount paper and the sample paper using the average step measurement tool. The measurements were carried out from five randomly selected points across the sample surface.

#### Water contact angle measurements for hydrophobicity detection of handsheet surfaces

The contact angles of distilled water on the untreated and tannin-treated handsheets were measured using a Contact Angle Meter Drop Master DMs-401 (Kyowa Interface Science Co., Ltd., Saitama, Japan). The droplet size was 0.5 µL, and the contact angle was calculated as the average of the left and right angles at 1-s intervals starting one second after the droplet was peeled off. The measurement time was 5 s. Two parallel measurements were performed for each treatment type (i.e., blank, TA2%, TA4%, TW2% and TW4%).

### Statistical analysis

Generalized linear mixed models (GLMMs) were used in statistical analyses using SAS Enterprise Guide 7.15 (SAS Institute, Inc., Cary, NC, USA). The explanatory variables (treatment type, i.e., tannin-rich bark extract (TW) vs. reference tannic acid (TA), square mass of paper (60 g/m^2^ vs. 130 g/m^2^), rate (i.e., concentration of the TA and TW solutions) and incubation time) for which levels were compared were used as fixed effects in the models. The second- and third-order interactions of these fixed effects were included in the models when possible. When a control treatment could not be classified as an explanatory variable, the models were simplified using only treatment as a fixed effect. The levels of fixed effects were compared by using Tukey’s pairwise comparison test with a significance level of α = 0.05. The assumption of unequal variances of the fixed effects was allowed when it improved the model (based on a value of AICC) and was accepted based on the likelihood ratio test (α = 0.05).

Correlated observations, such as time points, were modeled through a repeated effect with the assumption of compound symmetry (CS) covariance structure. Technical replicates were taken into account through a random effect instead of using just the mean of those. In the case of concentrations of tannic acid and bark extract, the measurements of a few time points were combined to simplify the statistical comparison of time points. The Kenward–Roger method was used to calculate the degrees of freedom. In almost all the models, the assumption of normal distribution was used for the response variables, but when a response variable (paper thickness) was heavily skewed, the assumption of gamma distribution with a log link was used. The assumption of normality of the residuals was studied graphically and was found to be adequate for all models.

## Results

### Chemical composition of the extract

Hot water extracted 59 mg/g of spruce bark. The yield of the purified extract was 31 mg/g bark dry matter. Qualified and quantified extracts were classified into different compound groups. The contents of the different compound groups in resin-treated hot-water extracts are presented in Table 1.

**TABLE 1:** Contents of the different compound groups in resin-treated TW extract. Condensed tannins were analyzed by UHPLC after thiolytic degradation of the tannins. Other compound groups were analyzed by GC. The content is expressed as mg/g dry extract and dry bark (mean ± stdev). The results are presented as mg/g per dry weight (DW) of extract and per DW of bark.

Compound group	mg/g DW (extract)	mg/g DW (bark)	
Stilbenes	59.80 ± 2.49	3.56 ± 0.19	
Organic acids	33.09 ± 1.58	1.97 ± 0.12	
Carbohydrates	26.22 ± 5.00	1.56 ± 0.31	
Flavonoids	27.35 ± 2.43	1.63 ± 0.16	
Alcohols	25.88 ± 1.25	1.54 ± 0.09	
Lignans	23.02 ± 2.82	1.37 ± 0.18	
Other aromatic compounds	6.57 ± 0.55	0.39 ± 0.04	
Fatty acids/derivatives	9.36 ± 0.97	0.56 ± 0.06	
Resin acids	3.98 ± 0.08	0.24 ± 0.00	
Steroids	2.06 ± 0.07	0.12 ± 0.01	
Terpenoids	0.90 ± 0.16	0.05 ± 0.01	
Organic salts	3.45 ± 0.40	0.20 ± 0.02	
Other	33.86 ± 22.52	2.02 ± 1.37	

Condensed tannins	mg/g DW (extract)	DP^a^	PC/PD^b^
	83.7 ± 1.50	5.82 ± 0.08	87/13

^a^ DP, average degree of polymerization.

^b^ PC/PD, proportion of procyanidins (PC) and prodelphinidins (PD).

The content of sugars and hemicelluloses of the crude extract was 305 mg/g DW. Treatment with XAD7HP decreased the content of these carbohydrates. Resin treated extract contained 188 mg/g DW (extract) sugars. Sugars both in the crude extract and in the XAD7HP–treated extract consisted mainly of glucose.

Dimeric stilbenoid species were also recognized and quantified in resin treated extract with HPLC. Two of them were assumably based on dimeric isorhapontin and two on dimeric astringin species. The content of these dimeric stilbenoids was 67 mg/g DW (extract). The majority of these were dimeric isorhapontin species consisting of 74 w% of dimeric stilbenoids. The presence of dimeric stilbenoids in spruce bark has been reported before (Li et al., 2008; Mikulski and Molski, 2010; Gabaston et al., 2017; Halmemies et al., 2021).

Condensed tannin (CT) content in the crude extract was 41.7 mg/g DW and it was doubled by the XAD7HP treatment (Table 1). The average degree of polymerization of CTs was 5.82. CTs were mostly made of (epi)catechins (i.e., procyanidins) with some (epi)gallocatechin moieties (prodelphinidins) agreeing with previous studies (Jyske et al., 2020a).

### Chemical composition of commercial tannic acid

Commercial tannic acid is a mixture of polyphenolic compounds and a product of natural origin. The content of tannic acids differs between producers, and even the separate lots of one supplier can contain different polyphenols (Salminen and Karonen, 2011). The tannic acid used in this study was characterized by UHPLC‒MS/MS: the polyphenols were mainly glucose-based gallotannins with some galloyl glucoses (see Supporting Information, Supplementary Figure S3).

### Antiviral efficacy of the tannin-rich bark extract (TW) and tannic acid (TA)

**FIGURE 2:**
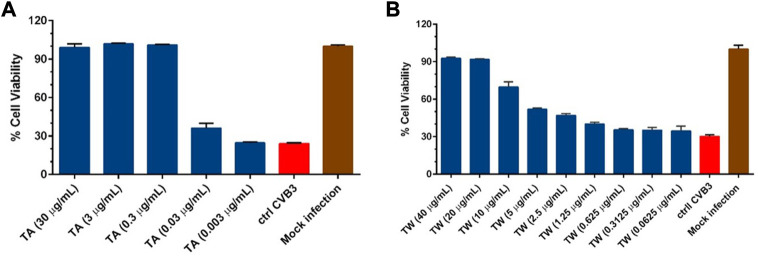
Testing the antiviral activity of **(A)** commercial tannic acid (TA) and **(B)** tannin-rich bark extract (TW) against enterovirus CVB3. Virus control and test samples are normalized against the mock infection. The results are the mean of three technical replicates. The results are expressed as average values + standard errors of the mean (SEM).

The antiviral potential of commercial tannic acid (TA) and tannin-rich bark extract (TW) was determined against the non-enveloped enterovirus CVB3. Here, the virus was pretreated with different non-cytotoxic concentrations of TA or TW for 1 h at 37°C. Many of the TA and TW concentrations showed antiviral activity by effectively inhibiting CVB3 infection (Figures 2A, B). In the case of TW, higher concentrations (40 μg/mL and 20 μg/mL) completely protected the cells from enterovirus infection. In addition, with the lowering of concentration values, their antiviral efficacy also decreased such that the lowest concentrations of the compound could not inhibit virus infection. A similar trend of decreasing antiviral efficacy with decreasing concentrations was also observed for TA. However, in the case of TA, a lower concentration of the compound (0.3 μg/mL) was successful in protecting the cells from CVB3 infection. These results indicated that TA showed slightly better antiviral activity than TW against CVB3. Here, we demonstrate the potent antiviral efficacy of both TA and TW to effectively inhibit enterovirus infection.

### Antibacterial efficacy of tannin-rich bark extract (TW) and tannic acid (TA)

**FIGURE 3:**
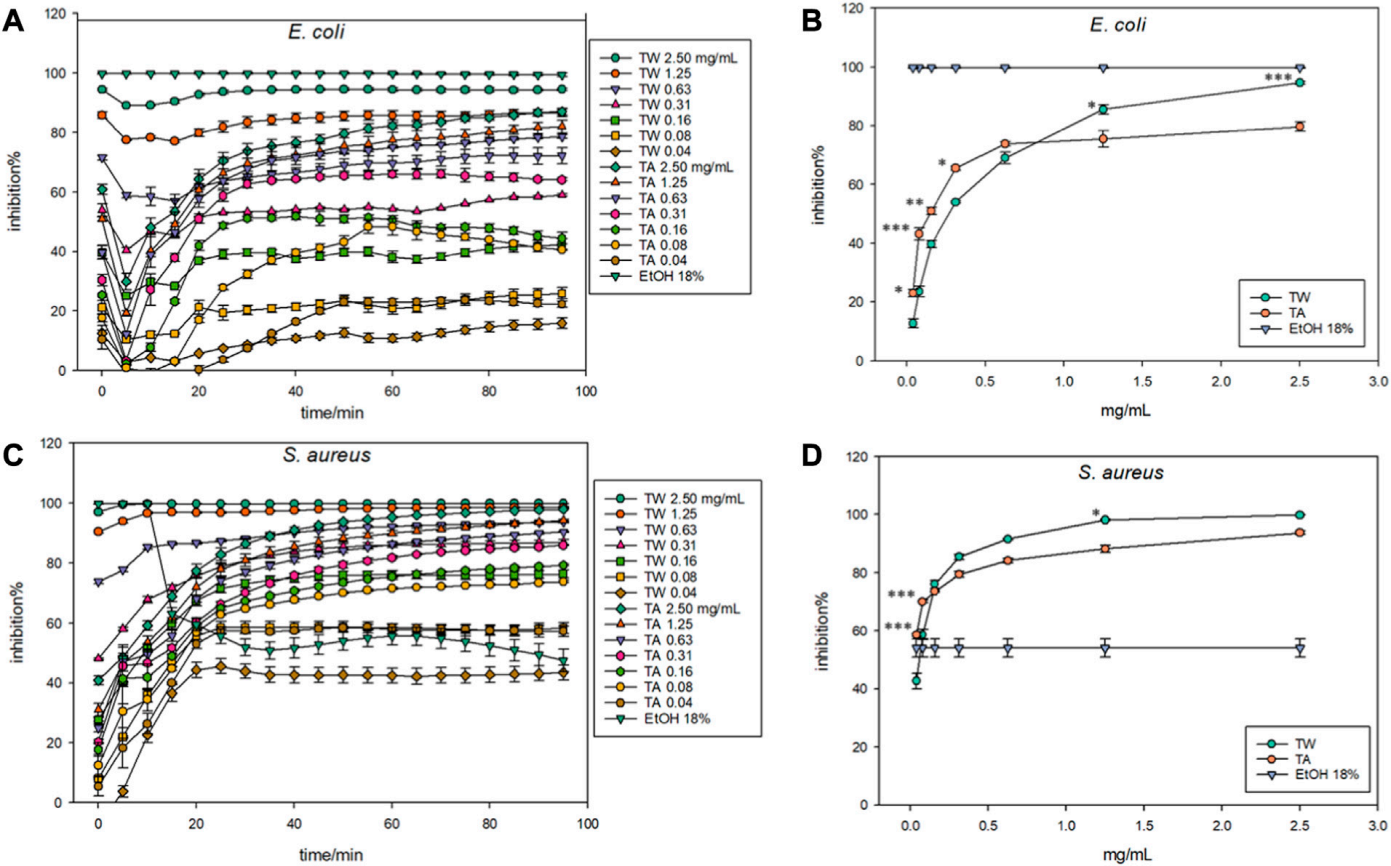
The bacterial biosensor luminescence production in inhibition percentages (inhibition%) in the presence of TW extract (TW) and tannic acid (TA) concentrations drawn against time in **(A)** (*E. coli*) and **(C)** (*S. aureus*) and drawn against the concentration in **(B)** (*E. coli*) and **(D)** (*S. aureus*), where the statistically significant differences between TW and TA concentrations are indicated with asterisks: *p ≤ 0.05, **p ≤ 0.01, ***p ≤ 0.001.

In Figure 3, we can see that all the TW and TA concentrations inhibit luminescence light production with both bacterial strains, especially after 20 min of incubation. Figures 3A, C show the behavior of the bacterial strains during the whole measurement and indicate that the luminescent light production is mostly stabilized after 20 min after adding the samples. By comparison of Figures 3B, D (at 50 min after the addition of samples), the *S. aureus* strain seems to be more sensitive toward the samples used than the positive control (18% ethanol), whereas *E. coli* is more sensitive to the dose of ethanol. With both strains, TW was more effective at higher concentrations: from 1.25 to 2.5 mg/mL for *E. coli* and from 0.16 to 2.5 mg/mL for *S. aureus*. However, at lower concentrations, tannic acid exhibited higher antibacterial activity against both strains.

With *E. coli* at 50 min of incubation (Figure 3B), all but the content of 0.63 mg/mL (*p* = 0.205) of TW and TA showed statistically significant differences. For *S. aureus* at the same time point (Figure 3D), only three of the extract contents of TW and TA showed statistically significant differences: 0.04 (*p* < 0.001), 0.08 (*p* = 0.002), and 1.25 mg/mL (*p* = 0.008), and an additional 0.63 mg/mL showed a marginally significant difference (*p* = 0.074). In addition, models, rate was used as a continuous variable. In particular, a logarithmic form of the rate correlated strongly with the concentrations; Pearson’s correlation coefficients were 0.884 and 0.719 for *E. coli* and *S. aureus*, respectively. Without log-transformation, the coefficients were 0.15 lower. These models resulted in similar results as the previous ones.

### Antiviral efficacy of TW- and TA-enriched handsheets

**FIGURE 4:**
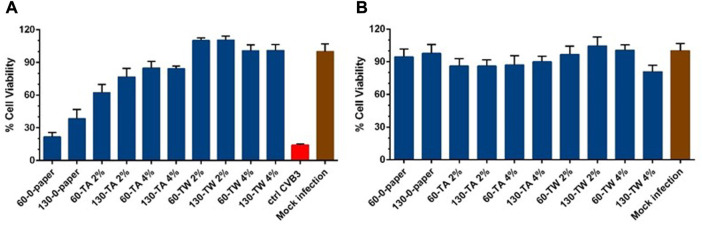
Testing the antiviral activity of paper samples against enterovirus coxsackievirus CVB3 **(A)** and cytotoxicity of paper samples on A549 cells **(B)**. Virus control and test samples are normalized against the mock infection. The results are the mean of two independent experiments. The results are expressed as average values + standard errors of the mean (SEM).

The antiviral activity of the paper samples was evaluated using the highly stable non-enveloped enterovirus CVB3. Here, we first tested the cytotoxicity of paper samples on A549 cells, as possible toxic components released from the materials to the cells could bias the possible antiviral outcome. Fortunately, the samples themselves were not toxic to the cells in the antiviral experiment setup that was used (Figure. 4).

Next, the antiviral activity was tested on paper surfaces where CVB3 was applied for a 5 min time interval at room temperature (Figure 4). When we tested the papers having no coating of any compound, we observed no apparent antiviral activity, especially on 60 g/m^2^ papers. The antiviral effect on the 130 g/m^2^ paper was higher but still modest, suggesting that heavier paper was better for antiviral action. The difference in the antiviral activity between the untreated 60 g/m^2^ and 130 g/m^2^ papers was, however, non-significant (*p* = 0.888). In the case of 60 g/m^2^ paper samples with TA coating, the antiviral activity increased with the increase in the amount of TA used for coating (4% TA coating was better compared to 2% TA), but this difference was not statistically significant (*p* = 0.645). When we tested 130 g/m^2^ paper samples coated with 2% and 4% TA, we did not observe any difference in the antiviral effect between the two (*p* = 0.999). The highest antiviral activity was observed in the paper samples coated with TW (i.e., the treatment effect was significant, *p* < 0.001). Moreover, the antiviral efficacy was similar between the papers of different thicknesses coated with TW (*p* = 1). When the significance of the main effects was analyzed, the main effect of treatment type was significant (*p* < 0.001), but that of concentration and handsheet thickness were not (*p* > 0.05). Moreover, an almost significant 2-way interaction between the treatment type and concentration in the antiviral activity was detected (*p* = 0.064). From Figure 4, it is evident that the antiviral activity was better in the paper samples of both thicknesses coated with 2% TW compared to those papers having the 2% TA coating (60 g/m^2^: TA2% vs. TW2%, *p* < 0.05; 130 g/m^2^: TA2% vs. TW2%, *p* < 0.05). However, no significant difference in antiviral activity was detected between the handsheets of both thicknesses treated with 4% concentrations of TA and TW (60 g/m^2^: TA4% vs. TW4%, *p* = 0.212; 130 g/m^2^: TA4% vs. TW4%, *p* = 0.186).

### Antibacterial efficacy of TW- and TA-enriched handsheets

**FIGURE 5:**
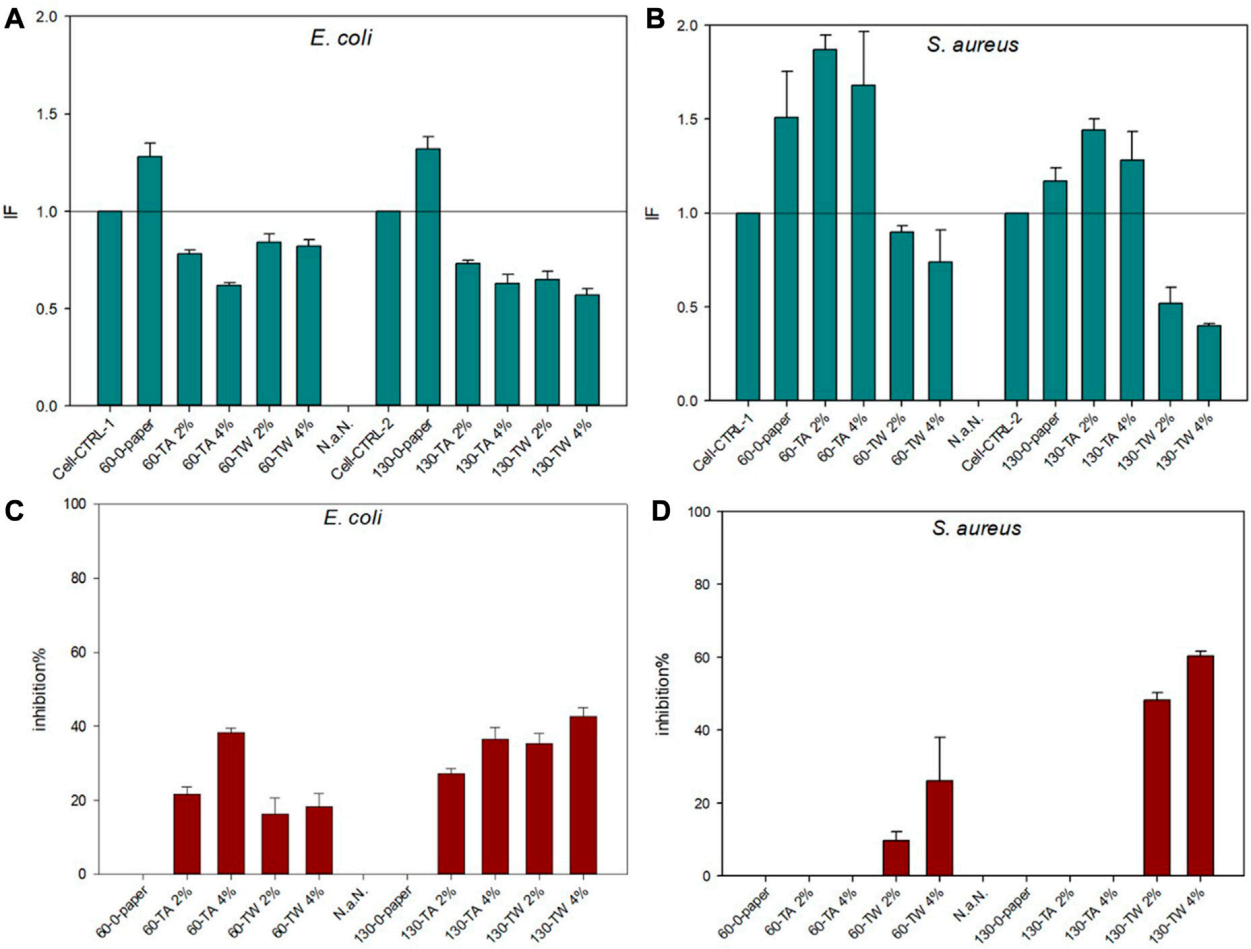
Bacterial imaging results in impact factors (IF) with *E. coli* **(A)** and *S. aureus* **(B)** and in inhibition% with *E. coli* **(C)** and *S. aureus* **(D)** for both paper thicknesses 60 g/m^2^ (60) and 130 g/m^2^ (130) and TW extract (TW) and tannic acid (TA) in two different doses 2% and 4%. The horizontal line in **(A)** and **(B)** indicates the level of impact factor (IF = 1) where no antibacterial effect is detected. Values below the horizontal line (IF = 1) indicate antibacterial effects by a decrease in luminescent light production, and values above indicate an increase in luminescence production. The error bars show the standard deviation of three replicates in separate Petri dishes. TA-enriched sheets create a clear inhibition zone in the *S. aureus* plates (Figure 6) but the light production is substantially increased at the zone border (Figure 7) likely because the killed bacterial cells provide nutrition to the survived ones.

**FIGURE 6:**
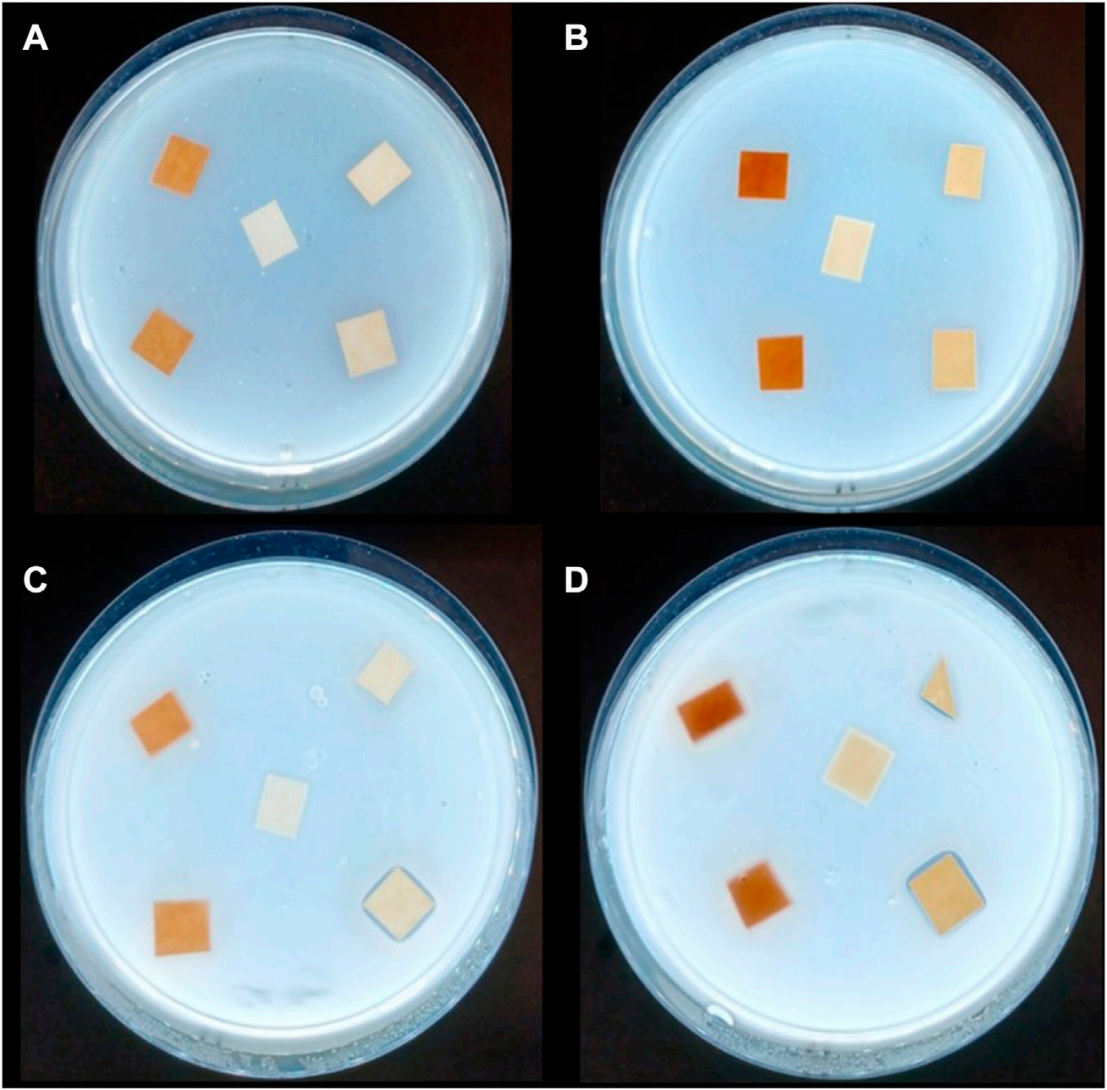
The sample papers in Petri dishes with *E. coli* **(A)** 60 g/m^2^ and **(B)** 130 g/m^2^ and *S. aureus* **(C)** 60 g/m^2^, and **(D)** 130 g/m^2^. Control or 0-paper is in the middle and others are in clockwise order starting from the upright: TA 2%, TA 4%, TW 2%, and TW 4% for all the plates **(A–D)**. Three identical Petri dishes were prepared for each sample set.

**FIGURE 7:**
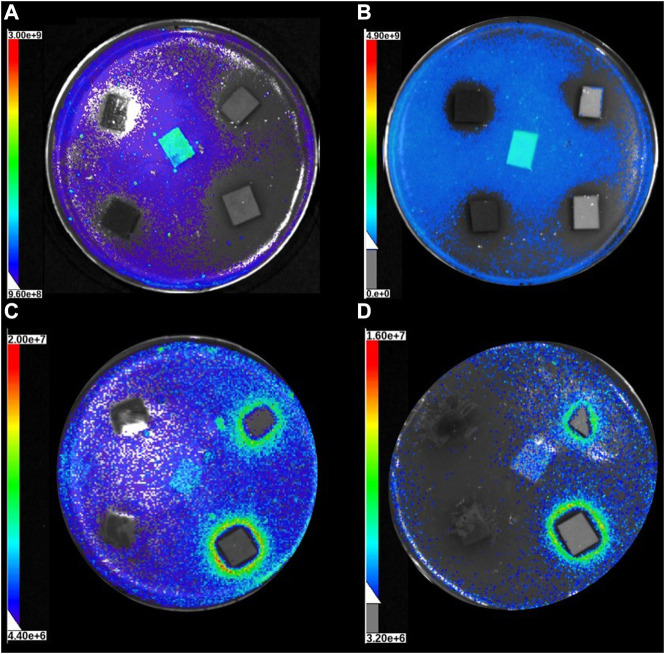
The sample papers in Petri dishes with *E. coli* **(A)** 60 g/m^2^ and **(B)** 130 g/m^2^ and *S. aureus* **(C)** 60 g/m^2^, and **(D)** 130 g/m^2^ imaged with the Lago *in vivo* imaging system in photo overlay mode. As in Figure 6, control or 0-paper is in the middle and others are in clockwise order starting from upright: TA 2%, TA 4%, TW 2%, and TW 4% for all the plates **(A–D)**. Three identical Petri dishes were prepared for each sample set, and the color scaling on the left of each plate indicates the linear luminescence light scaling in units of radiance.

Figure 5 shows the results from the bacterial imaging. With the *E. coli* strain, both sheet thicknesses and both doses of TA and TW inhibit luminescence light production, and the effect is dose dependent. However, it seems that inhibition is higher in 130 g/m^2^ paper with all extract additions except 4% tannic acid, where there is no significant difference with the 60 g/m^2^ paper with the same dose and addition. The main difference with *S. aureus* strain results is that with this strain, the luminescence light production increases in the sheets containing tannic acid doses, and only the sheets containing TW extract decrease the light production dose-dependently. The reason for the TA induced increase in the luminescent light production is indicated in the Figures 6, 7.

With *S. aureus* bacteria, there was a statistically significant difference between both TA vs. TW addition (*p* = 0.004 for TA60 vs. TW60 and *p* = 0.005 for TA130 vs. TW130) and paper thickness (*p* = 0.047 for TA60 vs. TA130 and for TW 60 vs. TW130). No significant differences were found in *E. coli* results in addition (*p* = 0.33 for TA60 vs. TW60 and *p* = 0.57 for TA130 vs. TW130) or paper thickness (*p* = 0.79 for TA60 vs. TA130 and *p* = 0.11 for TW 60 vs. TW130). The untreated 0-paper sample differed from all the treated papers for *E. coli* (*p* < 0.05) but not for *S. aureus* (*p* > 0.14 for all).

Figure 6 clearly shows that a clear zone of inhibition formed around the highest tannic acid concentrations with *S. aureus* (Figures 6C, D). Interestingly, light production was considerably increased at the inhibition zone border (Figures 7C, D), implying that something could leak from the sheet and that the *S. aureus* strain is able to be used as nutrition. The increase in light production could also be caused by surviving individual cells of the *S. aureus* strain being able to use killed bacterial cells as a source of nutrition in the inhibition zone border. No such phenomenon was observed with the *E. coli* strain sheets with tannic acid or the strain sheets with TW extract (Figures 6, 7). Instead, in Figure 7, the light production was visibly inhibited near the TW samples, and the effect was strongest with the thicker paper sheets (130 g/m^2^) (Figures 7B, D).

### Surface properties and topochemistry of tannin-enriched handsheets

#### Laser microscopy on surface roughness and thickness

**FIGURE 8:**
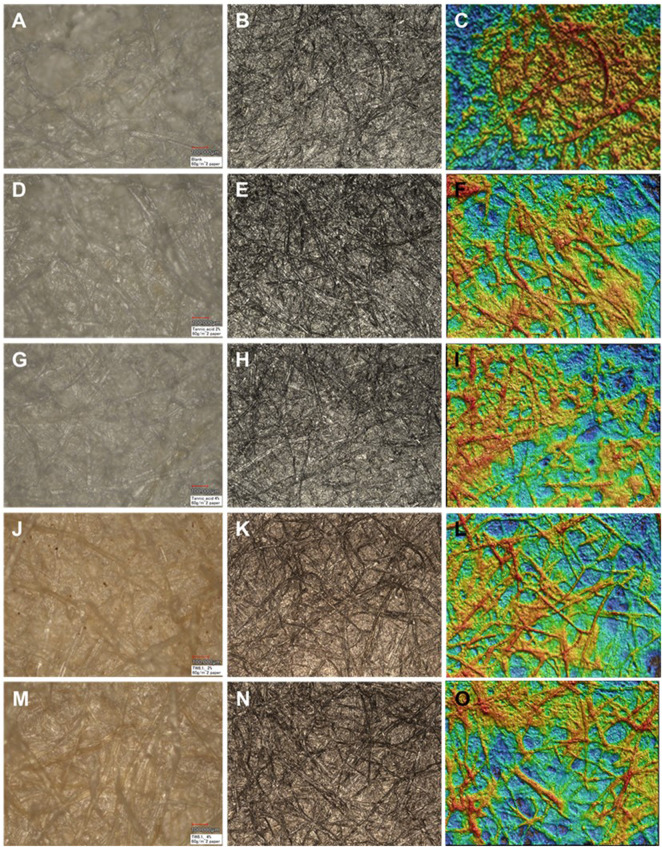
Laser microscopy of 60 g/m^2^ handsheets: control **(A–C)**, tannic acid (TA; **(D–F)** 2%; **(G–I)** 4%), and TW extract (**(J–L)** 2%; **(M–O)** 4%). The left side column of the subfigures represents opticalmicrographs; the center columns of the subfigures represent the laser image with color of the Extended Depth of Focus program; and the right-hand column of the subfigures represents the laser images as a 3D image.

**FIGURE 9:**
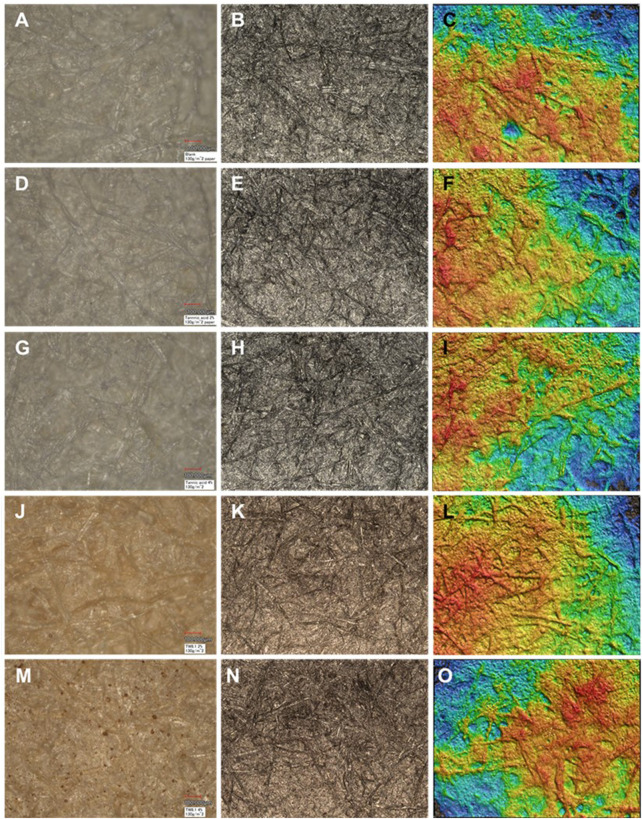
Laser microscopy of 130 g/m^2^ handsheets: control **(A–C)**, tannic acid (TA; **(D–F)** 2%; **(G–I)** 4%), and TW extract (**(J–L)** 2%; **(M–O)** 4%). The left side column of the subfigures represents opticalmicrographs; the center columns of the subfigures represent the laser image with color of the Extended Depth of Focus program; and the right-hand column of the subfigures represents the laser images as a 3D image.

**FIGURE 10:**
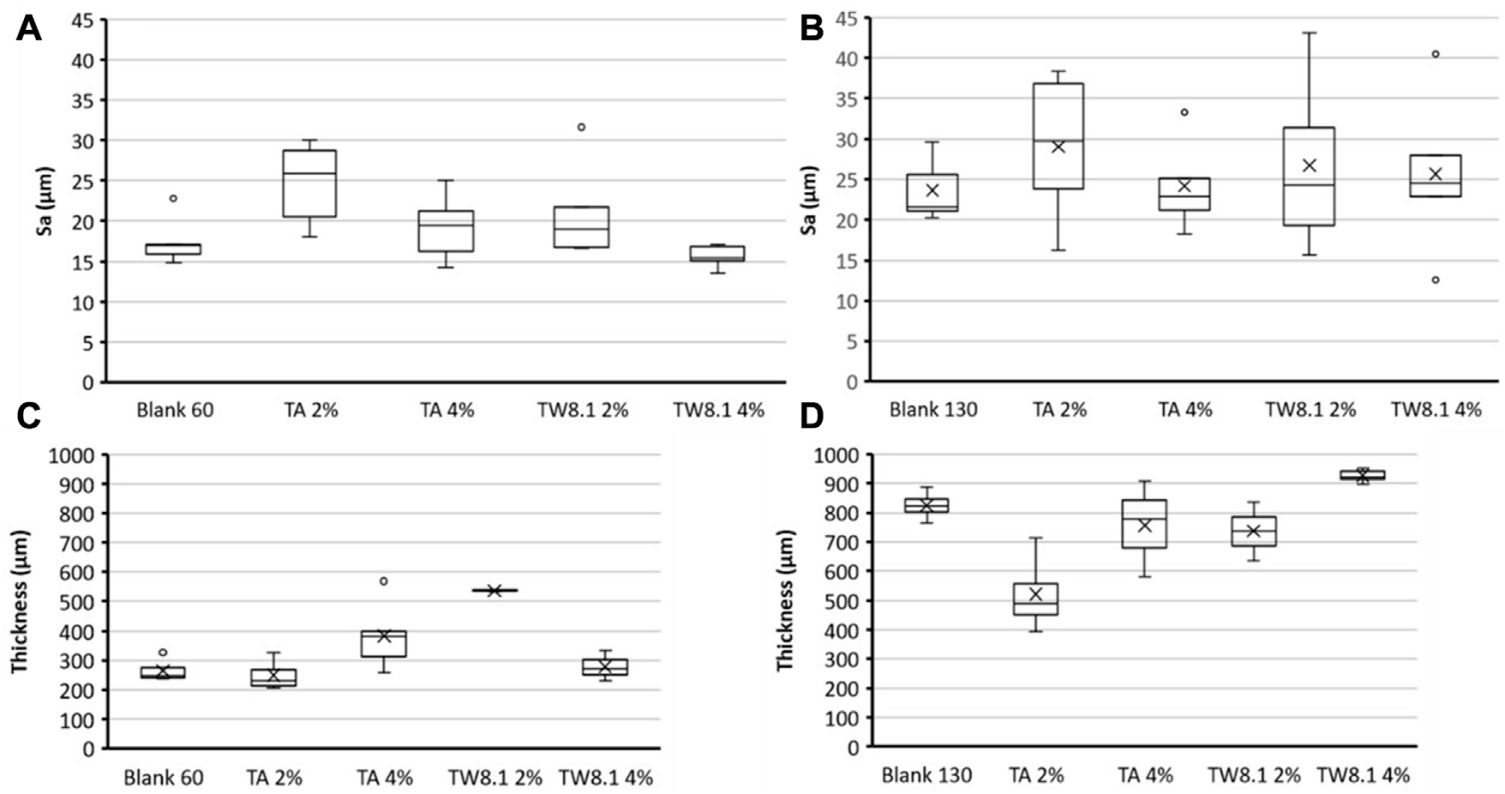
Properties of 60 g/m^2^ **(A, C)** and 130 g/m^2^ **(B, D)** handsheets treated with tannic acid (TA; blank control, 2% and 4%) and tannin-rich bark extract (TW; blank control, 2% and 4%): surface roughness **(A, B)** and sample sheet thickness **(C, D)**.

According to the laser scanning microscopy imaging of the paper samples, both the tannic acid and tannin extract TW were evenly spread on the fiber matrices of 60 g/m^2^ handsheets, except that of the 2% TW extract, which formed visible dark clusters (Figure 8), and the 4% TW, which showed clustering on the denser handsheet type of 130 g/m^2^ (Figure 9). The clustering may also have been reflected in the sample thickness measurements and surface roughness parameter, as seen in Figure 10. According to the statistical analysis, the roughness was slightly higher in the TW-treated samples of 130 g/m^2^ compared to that of 60 g (*p* < 0.08). Similarly, both the thickness of TA- and TW-treated samples were slightly higher in 130 g than in 60 g/m^2^ sheets (*p* < 0.09).

#### Water contact angle for hydrophobicity

**FIGURE 11:**
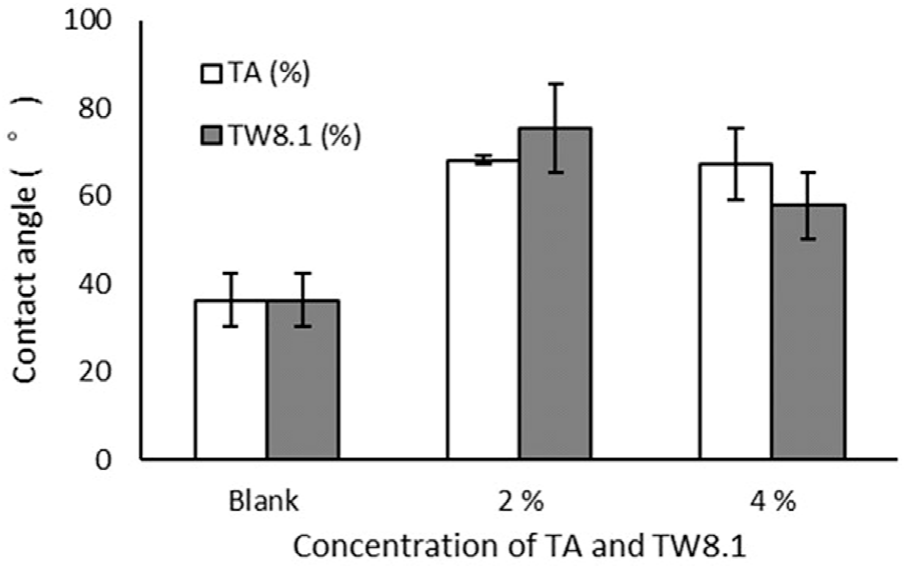
Water contact angles of handsheets treated with tannic acid (TA; blank control, 2% and 4%) and tannin-rich bark extract (TW; blank control, 2% and 4%). The measurements of 60 g/m^2^ and 130 g/m^2^ papers were pooled together (mean ± stdev).

The contact angle of the blank, untreated handsheets was 36.3° (Figure 11). The hydrophobicity of the handsheets significantly improved after both the TA (on average, 67.7°) and TW (on average, 66.6°) treatments compared to the blank specimens (*p* < 0.05). However, the TA and TW treated samples didn’t differ from each other, nor did the treatment concentrations of 2% and 4% of either TA or TW (Figure 11). The contact angle of uncoated handsheets dropped to 0° after 3 s from when the water droplet was placed on the specimen surface. In the coated samples, the water droplets became undetectable after approximately 5 s from the moment of absorption.

#### TOF-SIMS analysis of compound distribution

**FIGURE 12:**
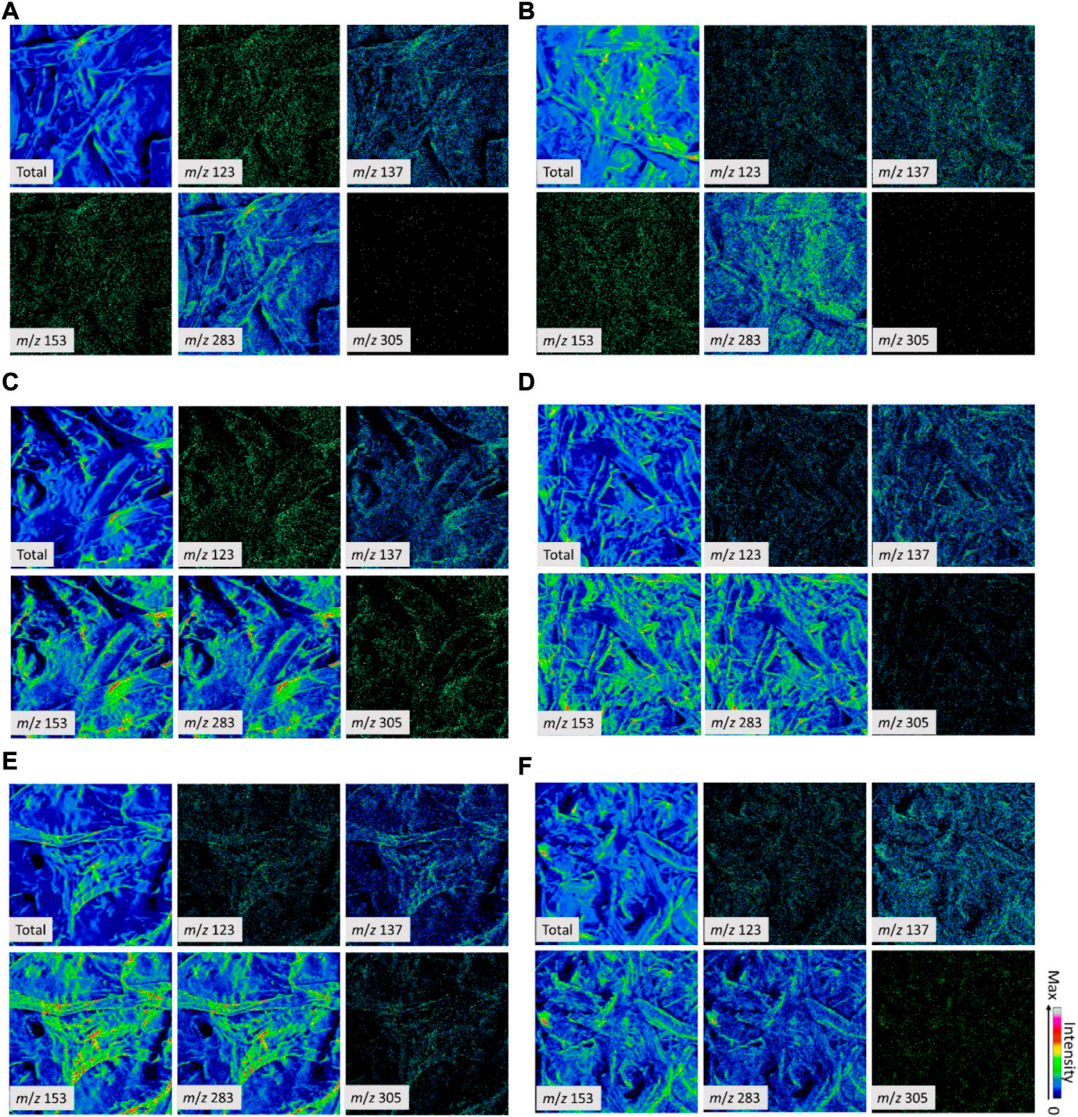
Positive ToF-SIMS images of the non-treated and tannic acid (TA)-treated paper samples of 60 g/m^2^ **(A, C, E)** and 130 g/m^2^ **(B, D, F)**. The peaks generated from tannic acid (*m*/*z* of 153 and 305), the surface of handsheets (*m*/*z* of 137 and 283), and a candidate peak from tannin-rich bark extract that was not present in the samples (*m*/*z* 123). Total secondary ions (Total) represent the surface structures of fiber matrices. **(A, B)**: blank; **(C, D)**, 2% TA; and E-F, 4% TA. The color scale was adjusted to 0–255 (8 bit) (bottom, zero; top, max).

**FIGURE 13:**
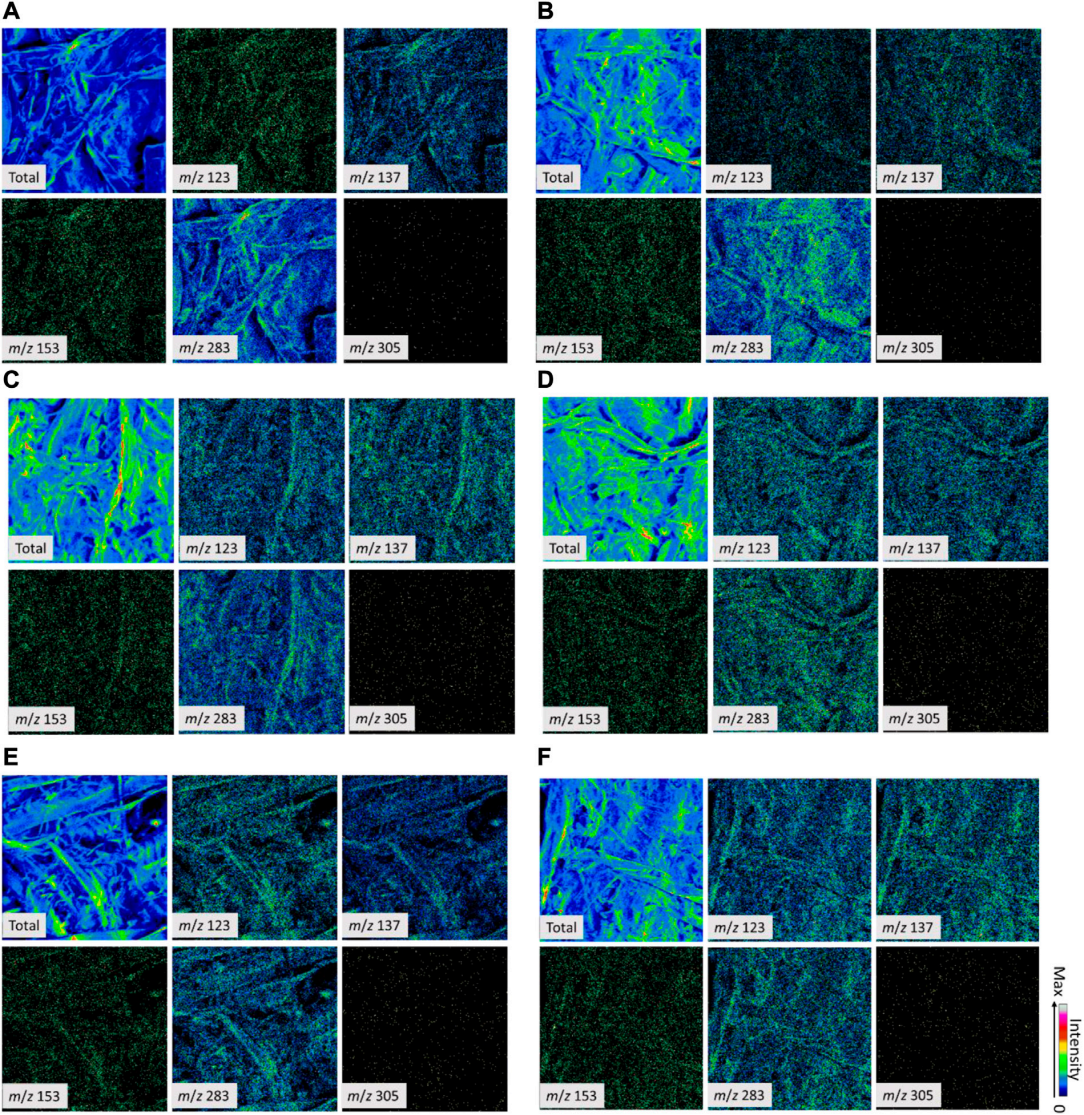
Positive ToF-SIMS images of the non-treated and tannin-rich bark extract (TW)-treated paper samples of 60 g/m^2^ **(A, C, E)** and 130 g/m^2^ **(B, D, F)**. The peaks generated from TW acid (*m/z* 123), the surface of handsheets (*m/z* 137 and 283), and candidate peaks from tannic acid were not present in the samples (*m/z* 153 and 305). Total secondary ions (Total) represent the surface structures of fiber matrices. **(A, B)**: blank; **(C, D)**, 2% TW; and E-F, 4% TW. The color scale was adjusted to 0–255 (8 bit) (bottom, zero; top, max).

First, we detected the spectra (both spectral and image-mode data) of the reaction products of tannic acid (TA) and freeze-dried extract (TW) (Supporting Information, Supplementary Figures S1, S2). Second, based on this analysis, candidate peaks were selected to be used to make images for identification of the final candidate peaks for the distribution analysis of tannic acid and TW on the paper samples (Supporting Information, Supplementary Tables S1, S2). For commercial tannic acid, *m/z* values of 153 and 305 for positive spectra and 124 for negative spectra were selected (Supplementary Figure S1). For freeze-dried TW, *m/z* 123 of positive and 124 of negative spectra were selected (Supplementary Figure S2). After comparative analysis based on these candidate peaks and other spectral data from the treated and non-treated paper samples, *m/z* 137 and 283 of the positive spectra and *m/z* 109, 136, and 255 of the negative spectra were selected as peaks from the handsheet itself (Supplementary Tables S1, S2). Note that *m/z* 124 of the negative spectra was detected for both tannic acid and TW. Based on the analysis, tannic acid and TW were evenly distributed on the papers (Figures 12, 13; Supplementary Figures S4, S5). Here, we must also note that in SIMS, the energy of the primary ion beam is high (in this study, 22 keV Au^+^ ions were used for positive ionization) in comparison to the covalent bond energies of the tannins, resulting in their fragmentation. In general, oligomeric and polymeric forms of condensed tannins are easily fragmented into smaller ones, even when soft ionization techniques, such as electrospray ionization, are used (Gu et al., 2003). Therefore, it is presumable that tannins are detected as low-molecular weight fragment ions and not as original intact molecular ions.

#### FTIR-ATR spectroscopy on potential chemical bonds

**FIGURE 14:**
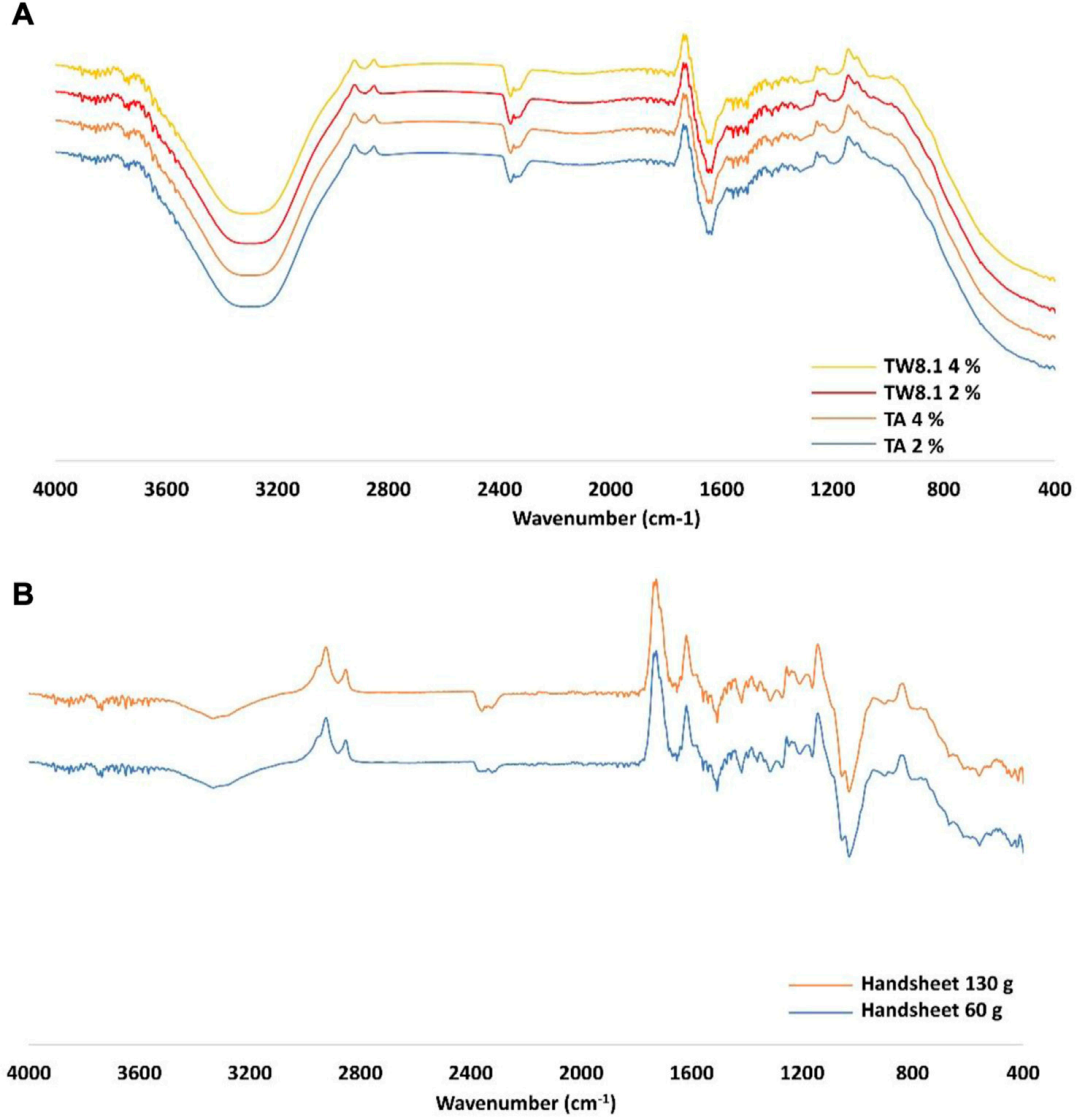
FTIR spectra comparison of **(A)** tannic acid (TA) and TW extract and **(B)** 60 g/m^2^ and 130 g/m^2^ handsheets.

As expected and shown in Figure 14, the FTIR-ATR spectra of the TA and TW extracts (both 2% and 4% extracts) had similar spectra due to the same kind of functional groups in the samples. In addition, the 60 g/m^2^ and 130 g/m^2^ handsheets without treatment (control samples) and accordingly, the 60 g/m^2^ and 130 g/m^2^ handsheets treated with 2% and 4% TA and TW extract, respectively, are handled in this interpretation as similar samples.

The handsheets (60 g/m^2^ and 130 g/m^2^) with and without extract treatment showed a broad weak band at circa 3,300 cm^-1^ attributed to O-H stretching vibrations for cellulose. In earlier studies, it has been reported that the intensity of the band is related to the number of functional groups, indicating more free OH groups and possibilities for inter- and intramolecular hydrogen bonds (Guan et al., 2009; Fan et al., 2012). In this study, some of the extract-treated handsheets showed slightly stronger bands at 3,300 cm^-1^ than untreated sheets, as well, but the results were not unambiguous. The sheets with or without the extract treatments showed a band at approximately 2,880 cm^-^1 attributed to the C-H stretching vibrations of the hydrocarbons of the polysaccharides. Several characteristic weak peaks of cellulose are found at 1,680–900 cm^−1^, for example, at 1,489, 1,474, 1,396, 1,387, and 1,317 cm^−1^, due to the stretching and bending vibrations of the O-H, C-H, and C-O bonds of cellulose (Hospodarova et al., 2018). In addition, the bands at 1,055 and 1,029 cm^−1^ are attributed to the C-O stretching of polysaccharides (Dassanayake et al., 2018).

In the spectrum of TA (2% and 4%) and TW (2% and 4%) treated handsheets (60 g/m^2^ and 130 g/m^2^), shown in Supplementary Figures S6, S7, the characteristic weak peaks of tannic acid at 1716 cm^−1^ and 1,200–1,207 cm^−1^ contributed to the stretching vibrations of carboxylic ester ν(C=O) and ν(C-OH) extension in the aromatic rings of tannins, respectively (Falcão and Araújo, 2018). The ν(C-OH) vibration relates to the characteristic δas(O-H) bending vibration at 1,458 cm^−1^, as well. The C=C vibrations of aromatic rings of tannins are shown very weakly but characteristically in the spectral area of 1,600–1,400 cm^−1^, exactly at 1,576 cm^−1^, 1,541 cm^−1^, 1,518 cm^−1^, and 1,437 cm^−1^ (Falcão and Araújo, 2018).

## Discussion

The coronavirus disease (COVID-19) pandemic caused by SARS-CoV-2 has caused ca. Six million direct deaths (between 1 Jan 2020, and 31 Dec 2021), and more than 18 million people have died as measured by the excess mortality deaths worldwide (Wang et al., 2022). It is already known that coronaviruses will mutate and cause outbreaks in the future as well. In addition, the WHO lists that, for example, Ebola, Marburg, Lassa fever, MERS-CoV, SARS, Nipah, and Zika may cause significant outbreaks in the future (World Health Organization WHO, 2023). Furthermore, according to a WHO report from 2022, in the European Union or European Economic area alone, more than 670,000 infections are caused by antibiotic-resistant bacteria, and approximately 33,000 people die annually as a direct consequence of these (World Health Organization WHO, 2002). Thus, sustainable approaches for mitigation and infection control are needed to prevent damage caused by viral and microbial outbreaks in the future for societies.

This study provides baseline knowledge and proof-of-concept for the further development of antimicrobial, biobased functional coatings, and protective materials by valorizing the side streams of forestry industries, focusing on the condensed tannin-rich extract of Norway spruce bark as a case study. Similar value-added phenolics can also be obtained from other wood species or plant biomasses, such as willows, as shown in our earlier study (Tienaho et al., 2021) and those by (Lohtander et al., 2020; Lohtander et al., 2021)

Broad-acting antimicrobial materials may be created by impregnating antimicrobial compound(s) to different materials or binding those compounds as a covalent coating on a material(s) to prevent viral transmission by or through the handled materials (Kaczmarek, 2020). Research exists on the use of tannic acid in different materials (Halim et al., 2018; Ferreira Leite et al., 2021; Ulu et al., 2021), but to the best of our knowledge, no studies have been conducted on utilizing the condensed tannin-rich extract of Norway spruce to produce broadly acting antimicrobial tissue and paper materials (i.e., evidence against both virus and bacteria).

As the invented natural antimicrobials may possess unknown threats for health or nature, the preferred approach for getting the product to the market is to apply covalent binding of the active molecules on the desired surface material to minimize regulatory burden related to getting a product launched to the market. In addition, while the molecular stability on the coated surface remains unknown, the preferred applications are compostable/recyclable, single-use (disposable) materials. However, depending on the safety related to the discovered molecules, the use of the molecules as active/non-coated ingredients in other application fields may offer significant commercial opportunities.

The growing market segments are, e.g., the personal protective equipment products market ($92.86 Billion by 2027 (CAGR 7.4%), the antimicrobial packaging market for food applications [$13.28 Billion by 2027 (CAGR 5.4%)], and sterile and antiviral packaging market for medical products [$30 billion by 2026 (>5% CAGR)] (European Commission, 2020). All the mentioned markets offer lucrative growth opportunities for novel and innovative biobased/biodegradable antimicrobial products.

According to our results, the condensed tannin-rich extract obtained from industrial Norway spruce bark by hot water extraction was active against both Gram-negative and Gram-positive bacteria as well as against Enterovirus coxsackievirus B3. Enteroviruses are non-enveloped viruses that are typically more resistant to extreme pH, heat, dryness, and simple disinfectants than enveloped viruses (such as coronaviruses). Additionally, a commercial reference, tannic acid, was shown to have antimicrobial activity against enterovirus B3 as a solution in our studies. Furthermore, several earlier studies have shown that tannic acid has antiviral activity against different viruses (Konowalchuk and Speirs, 1975; Kaczmarek, 2020), and botanic tannic acid-derived clinical therapy has shown activity against two coronaviral strains, SARS-CoV-2 and HCoV-OC43, which are the earliest-known coronaviruses (Shih et al., 2022). According to previous literature, antibacterial and antibiofilm activity has also been reported from tannic acid (Dong et al., 2018; Jing et al., 2022). Chung et al. (Chung et al., 1993) described the growth inhibition of tannic acid against 15 different bacterial cells, including *E. coli* and *S. aureus*. Tinto et al. (Tintino et al., 2016) also showed that tannic acid has a synergistic effect against *S. aureus* when associated with antibiotics. Jing et al. (Dong et al., 2018) suggested and reported various potential mechanisms of action for tannic acid, and one of these would be its capability to directly bind to the peptidoglycan of the bacterial cell walls, which interferes with the cell wall integrity.

Thus, we could confirm our first hypothesis that condensed tannin-rich bark extract as well as a commercial reference, tannic acid, possess broad spectrum antimicrobial activity against both bacteria and the tested virus.

Our results indicated that when the tannic acid and tannin-rich extract were immobilized into fiber handsheets by dip coating, their antimicrobial activity remained against both the tested bacteria and the virus. The antibacterial activities of the handsheet samples were investigated by using Gram-negative *E. coli* and Gram-positive *S. aureus* bacterial strains. *E. coli* and *S. aureus* have been reported as the leading pathogens of health care-associated infections and bacteremia, especially in elderly individuals (Poolman and Anderson, 2018). The antiviral activity of the handsheet samples was evaluated using the highly stable non-enveloped enterovirus CVB3. Enteroviruses are responsible for causing acute infections such as meningitis, pancreatitis [reviewed in (Simons-Linares et al., 2021)] and myocarditis (Bouin et al., 2019). They are also associated with chronic infections such as type I diabetes [reviewed in (Nekoua et al., 2022)]. In addition, these non-enveloped viruses are resistant to disinfectants. Currently, there are no approved antivirals against enteroviruses on the market. Hence, it is important to tackle these viruses and reduce the viral load around us.

In this study, we used the dip coating method to study the potential of condensed tannins and tannic acid incorporation into the fiber network of lignocellulosic handsheets. During the dip coating method, the whole handsheet substrate was immersed into the tannin extract solution, and the solvent was evaporated. Our results from the topochemical ToF-SIMS and laser microscopy analysis indicated that the compounds of both tannic acid and bark extract were evenly spread and localized in the fiber matrices. The hydrophobicity of the handsheet surfaces was also significantly higher after the TA (87%) and TW treatments (83%) compared to untreated samples.

No covalent chemical bonding between bioactive compounds and fiber surface molecules could be detected, as was to be expected since no additional chemical agents for binding or surface modification were applied. FTIR-ATR was used for surface molecular characterization and provided information about the characteristic chemical bonds of the cellulose substrate and the immersed TA and TW extracts. Although the characteristic area of tannins and tannic acid partly overlapped with the cellulose vibrations, all the characteristic peaks of extract were found in the spectra of TA- and TW-treated handsheets. Chemical bonding between the extract and the surface was not observed. However, the FTIR-ATR results allow for speculation about the possible hydrogen bonding on the extract-treated specimens.

The widely used coating methods to inhibit the spread of infections include dip, spray, spin and cast coating, bacterial colonization, and biofilm formation on surfaces. In biomedical applications, polymer- and nanocomposite-based antimicrobial and antiviral surface coatings have been prepared by using chemical surface modifications so that the coating agent is able to be bound covalently onto the surface (Balasubramaniam et al., 2021; Erkoc and Ulucan-Karnak, 2021). Adhesion between the surface and the coating may occur by mechanical interlocking via topographical modifications of the surface via van der Waals and electrostatic interactions, acid-base interactions and/or hydrogen bonding, but covalent bonding across the interface enables constant coating (Awaja et al., 2009). In a concurrent manner, the antibacterial and antiviral activities should be preserved in the final coated product. In addition to the extract composition and the selected binding method, the physical properties of the surface, such as porosity, hydrophobicity, topography, and virus absorption, affect the persistence of these bioactivities on the surface (Rakowska et al., 2021).

In future studies, we will focus on developing optimized binding chemistry for nature-derived antimicrobial mixtures applied to fiber matrices, including antimicrobial efficacy testing against a broader selection of bacteria and viruses (such as seasonal coronaviruses HCoV-OC43 and SARS-CoV2). Furthermore, to explore the structure-activity relationships of the components of CT-rich spruce bark extract and their antiviral/antimicrobial activities, we will concentrate on activity-guided fractionation of the extract and on the characterization of the components of the active fractions. Moreover, the comprehensive utilization concepts of bark side streams will be further studied, including techno-economic and business-model analysis of industrial scale-up possibilities of antimicrobial component production when integrated in circular bioproduction ecosystems (Rasi et al., 2019; Kilpeläinen et al., 2023).

## Data Availability

The original contributions presented in the study are included in the article/Supplementary Material, further inquiries can be directed to the corresponding author.
