# Supplementary material for: Inspired by nature: Fiber networks functionalized with tannic acid and condensed tannin-rich extracts of Norway spruce bark show antimicrobial efficacy
Source: Front Bioeng Biotechnol. 2023 Apr 19;11:1171908. doi: 10.3389/fbioe.2023.1171908 (PMC10154533; doi:10.3389/fbioe.2023.1171908)
Supplement: Supplementary file 1 [file Presentation1.pdf]

Supplementary Material

A

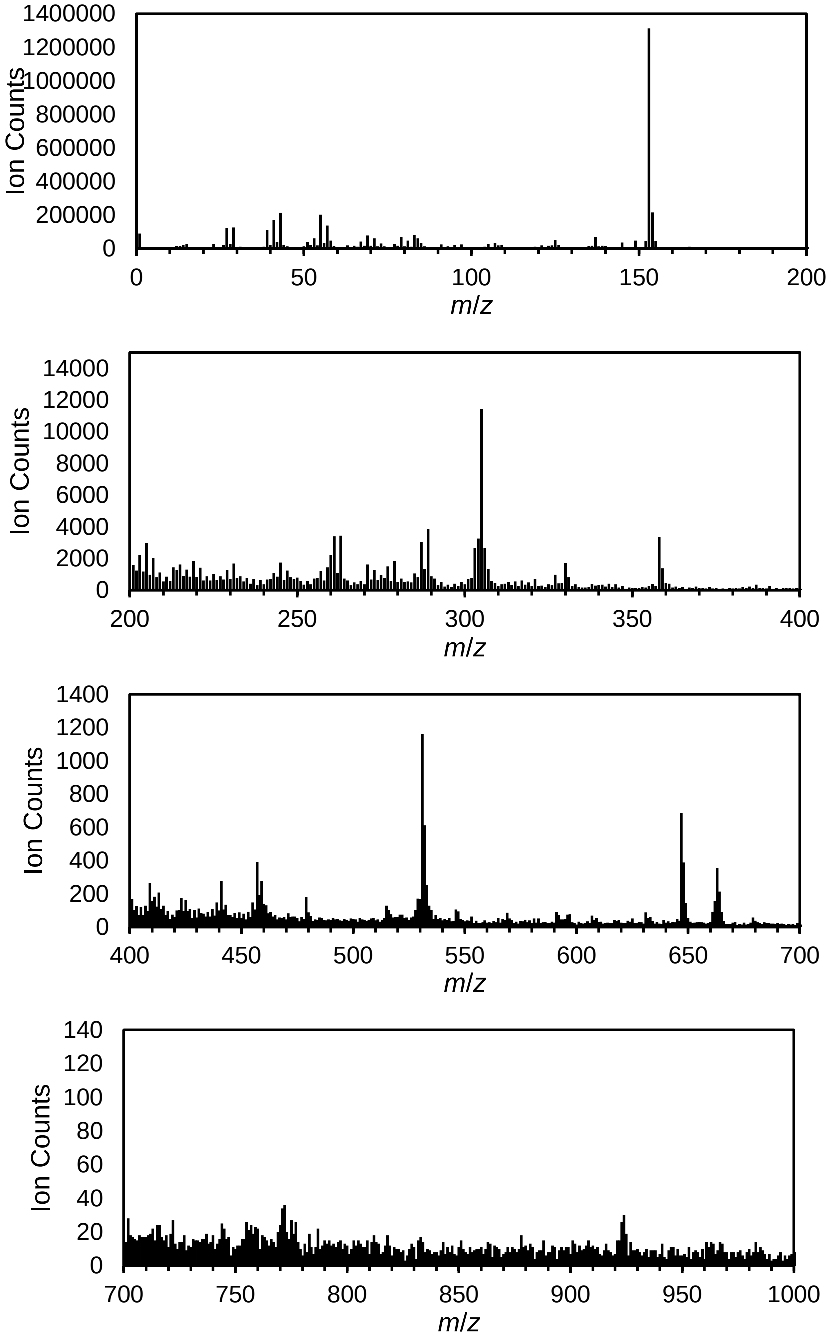

B

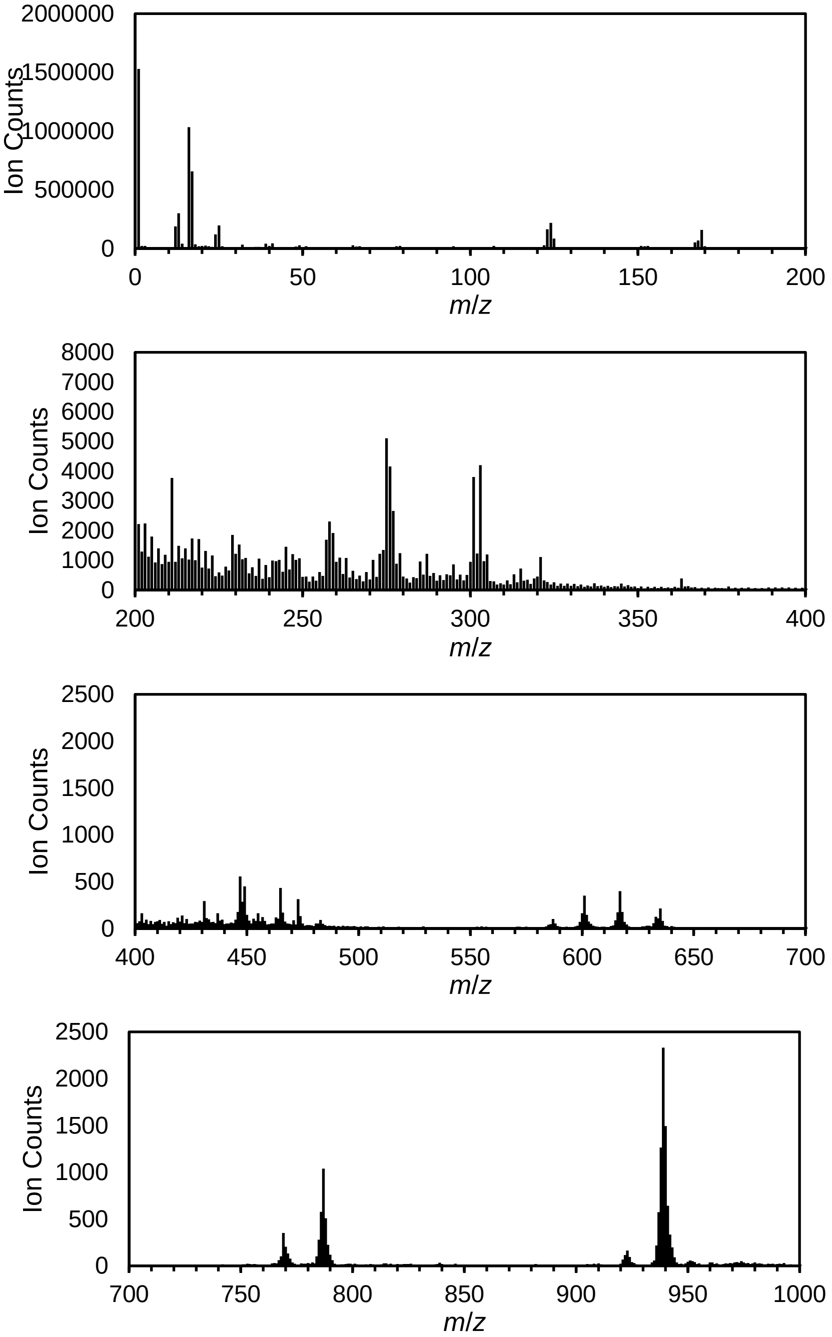

Figure S1. Positive (A) and negative (B) spectra of tannic acid (TA). The positive peaks generated from tannic acid (*m*/*z* of 153 and 305) were selected as candidate peaks for localization analysis of handsheets.

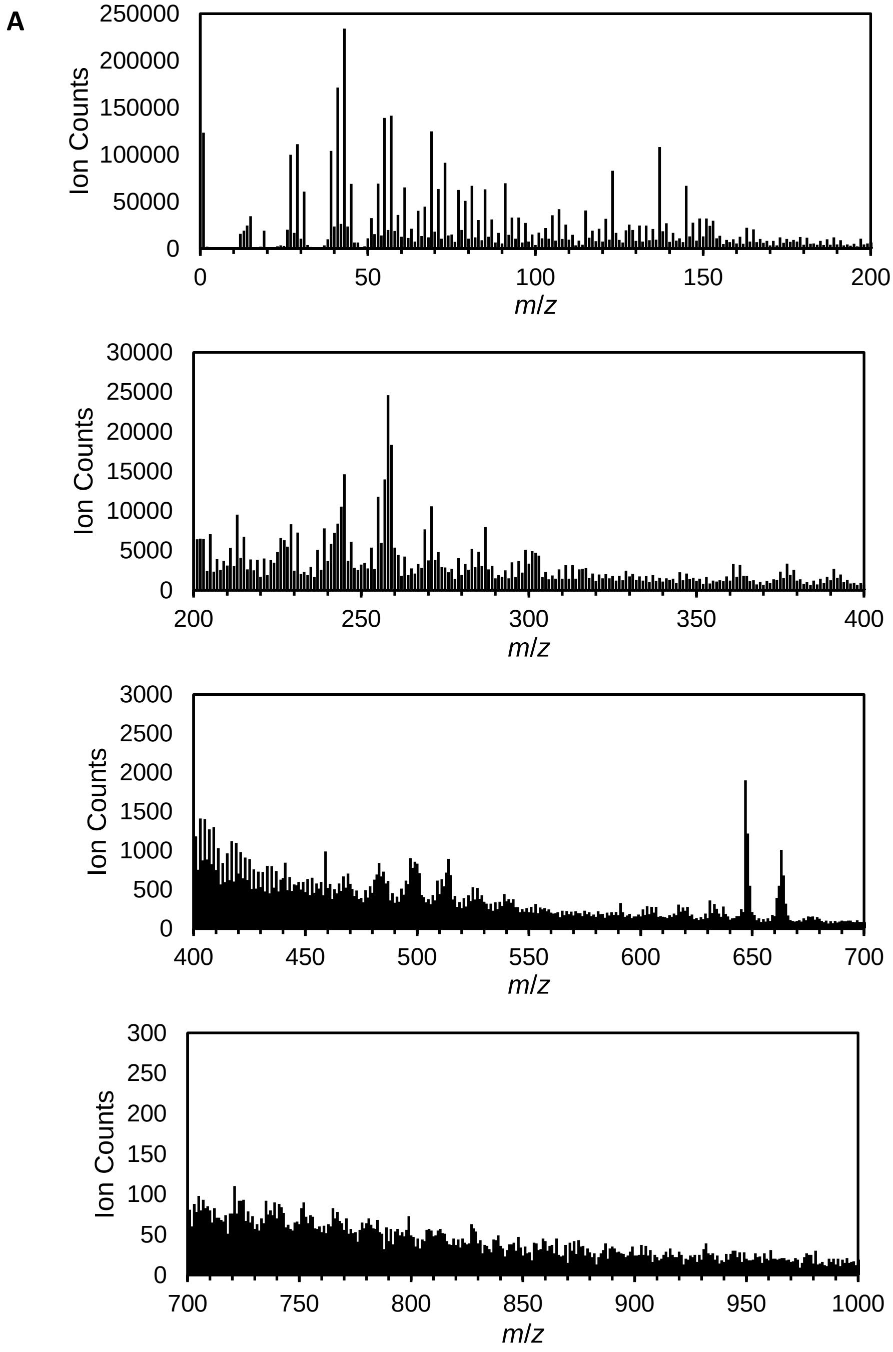
A
Ion Counts
m/z
0
50
100
150
200
250
300
350
400
450
500
550
600
650
700
750
800
850
900
950
1000
250000
200000
150000
100000
50000
30000
25000
20000
15000
10000
5000
3000
2500
2000
1500
1000
500
300
250
200
150
100
50

B

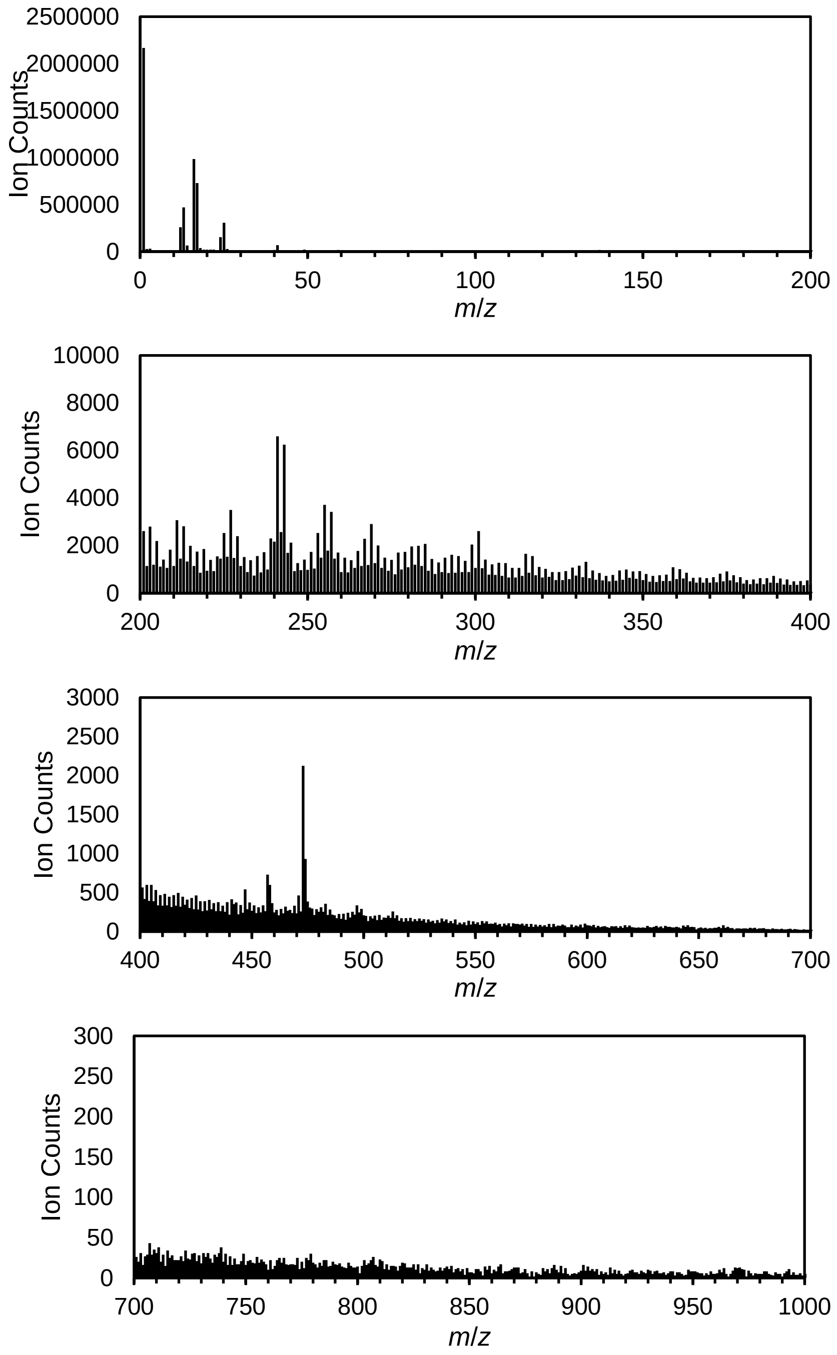

Figure S2. Positive (A) and negative (B) spectra of tannin-rich Norway spruce bark extract (TW8.1). The positive peak generated from extract (*m*/*z* of 123) was selected as a candidate peak for localization analysis of handsheets.

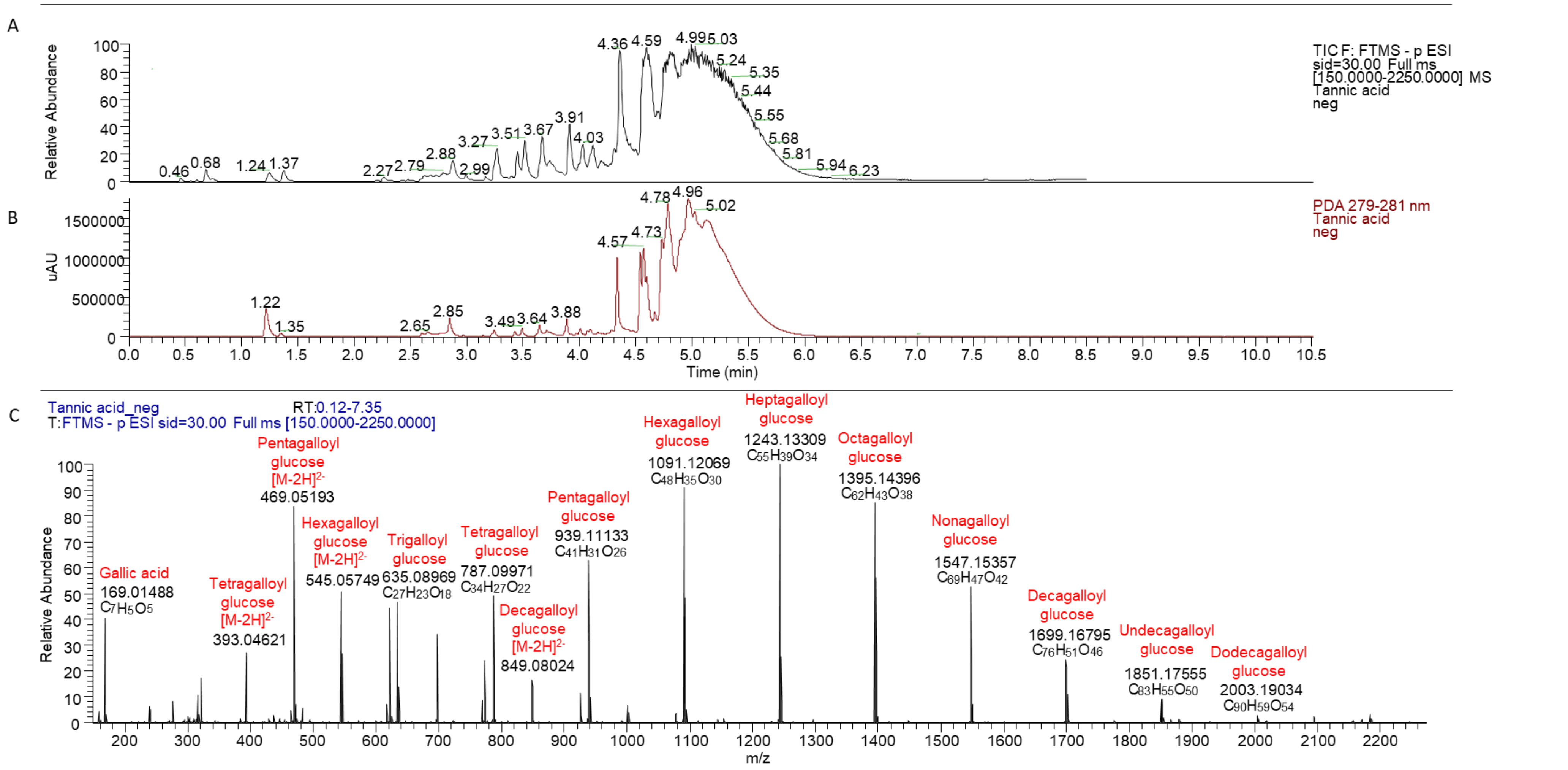

Figure S3. UHPLC-MS/MS total ion chromatogram in negative ion mode (A) and UV chromatogram at 279-281 nm (B) of tannic acid. The ultrahigh-resolution mass spectrum (C) was taken over the whole chromatogram length (RT = 0.12 – 7.35 min) and represents the most intensive ions detected. Ions identified are $[M-H]^-$ ions unless otherwise indicated.

Table S1. Detected positive candidate spectra of tannic acid (TA) and tannin-rich extract (TW8.1) in paper handsheets of 60 g/m^2 and 130 g/m^2.

Positive	1<br>Paper<br>60 g	2<br>Paper<br>130 g	3<br>Tannic acid 2%<br>60 g	4<br>Tannic acid 2%<br>130 g	5<br>Tannic acid 4%<br>60 g	6<br>Tannic acid 4%<br>130 g	7<br>TW8.1 2%<br>60 g	8<br>TW8.1 2%<br>130 g	9<br>TW8.1 4%<br>60 g	10<br>TW8.1 4%<br>130 g
99	+++	+++	+++	+++	+++	+++	+++	+++	+++	++
123		+					+++	+++	+++	+++
137	++	++	+++	+++	+++	+++	+++	+++	+++	+++
145	++	++	+		++	+++	+++	+++	+++	+++
153			+++	+++	+++	+++				
197	+	+	+++	+++	++	+	+	+	+	+
211	+	+	++	++	++	+	+			+
239	+++	++	+++	+++	+++	++	+	+	+	+
283	+++	+++	+++	+++	+++	+++	++	++	++	++
287	++	++	++	++	++	++	+++	+++	+++	+++
305			+++	+++	+++	+++				
381	++	++	++	+	+	+				
397	+++	+++	+++	+++	+++	+++		++		
411	+++	+++	++	+++	+++	+++	+	++	+	+
429	+++	+++	+++	+++	+++	+++		++		+
647			+		+		++			++

Table S2. Detected negative candidate spectra of tannic acid (TA) and tannin-rich extract (TW8.1) in paper handsheets of 60 g/m^2 and 130 g/m^2.

Negative	1<br>Paper<br>60g	2<br>Paper<br>130 g	3<br>Tannic acid 2%<br>60 g	4<br>Tannic acid 2%<br>130 g	5<br>Tannic acid 4%<br>60 g	6<br>Tannic acid 4%<br>130 g	7<br>TW8.1 2%<br>60 g	8<br>TW8.1 2%<br>130 g	9<br>TW8.1 4%<br>60 g	10<br>TW8.1 4%<br>130 g
101	+++	+++								
107	+	+	+++	+++	+++	+++	+	+	+	+
108	++	++	++	++	++	++	++	++	++	++
109	++	++	++	++	++	++	++	++	++	++
124			+++	+++	+++	+++	++	++	++	++
136	+++	+++	+	+	+	+	++	++	++	++
137	+	+	+	+	+	+	+++	+++	+++	+++
149	++	+					++	++	++	++
151	+++	+++	++	++	++	++	+++	+++	+++	+++
153	+	+	++	++	++	++				
161	+++	+++	+	+	+	+	+++	+++	+++	+++
162	+++	+++	+	+	+	+				
163	+++	+++	+	+	+	+	+++	+++	+++	+++
169			+++	+++	+++	+++				
171	+++	+++	++	++	+++	+++				
177	+++	+++	++	++	++	++	++	++	++	++
181	+++	+++	+++	+++	+++	+++				
183	+++	++	++	+++	++	++	+	+	+	+
211	+	+	+++	+++	+++	+++	+	+	+	+
237	+++	+++	+++	+++	+++	+++				
255	+++	+++	+++	+++	+++	+++	+++	+++	+++	+++
269	+++	+++	+	+	+	+	++	++	++	++
275			+++	+++	+++	+++				
276			+++	+++	+++	+++				
301			++	++	++	++				
303			+++	+++	+++	+++				
305			+	+	+	+	++	++	++	++
311		+		+++	+	+			+	++
319	+++	+++	+	+	+	+		+++	+++	+
321			++	++	++	++				
325	+++	++	+	+++	+				+	+
339	+++	+++	+	+++	+	+				
367	++	++				+				
447			+++	+++	+++	+++				
473	++	++	+		+		++			++
601			++	+++	++	++				
617			++	++	++	++				
787			+++	+++	+++	+++				
939			+++	+++	+++	+++				

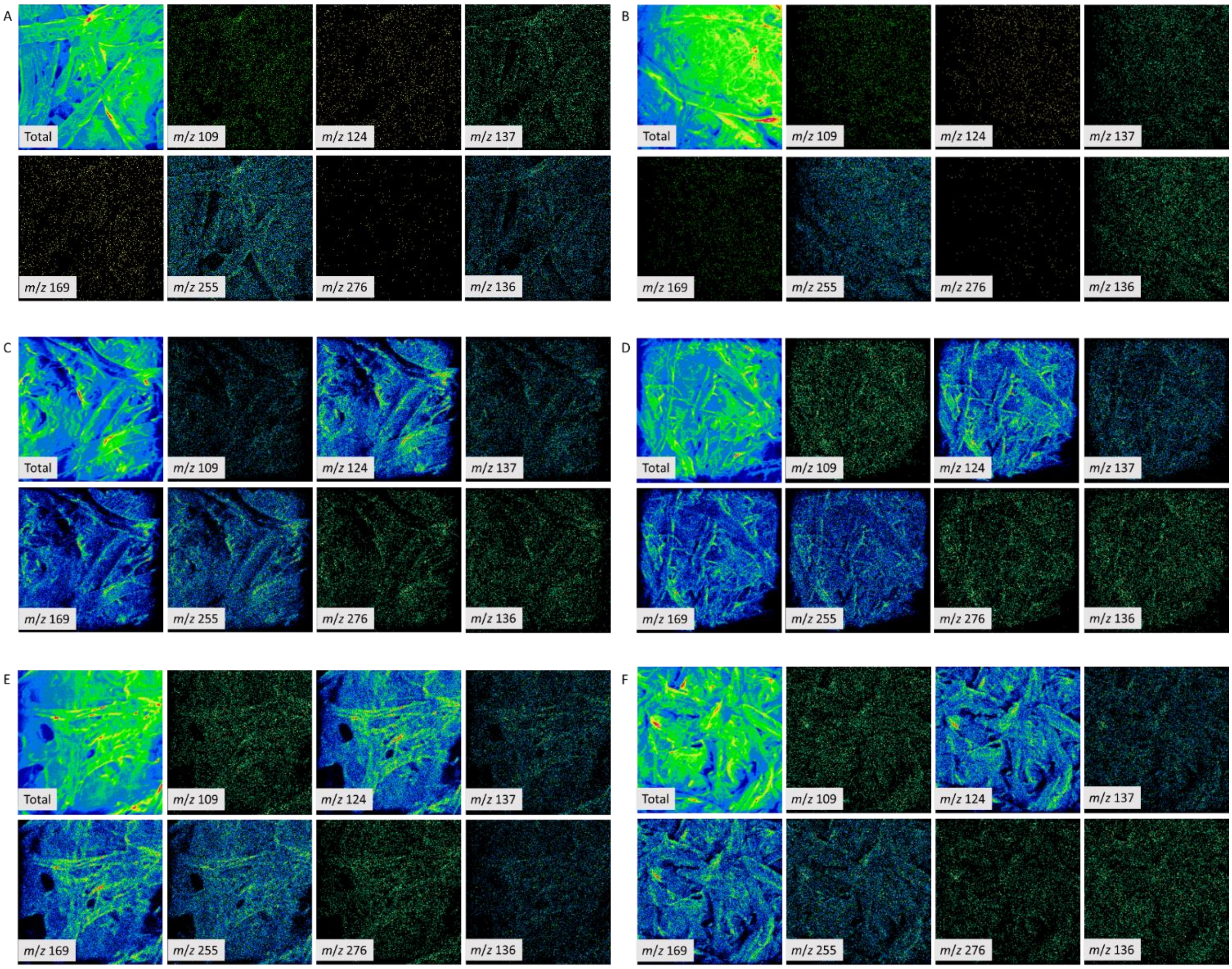

Figure S4. Negative ToF-SIMS images of the non-treated and tannic acid treated paper samples of 60 g/m^2 (A, C, E) and 130 g/m^2 (B, D, F). The peaks generated from tannic acid (*m/z* of 124), and surface of paper handsheets (m/z of 109, 136, and 255). Note, that the negative candidate peak from tannic acid (not present in the sample) that was also *m/z* of 124. Total secondary ions (upper left corner in each sub-figure) represent surface structures of fiber matrices.

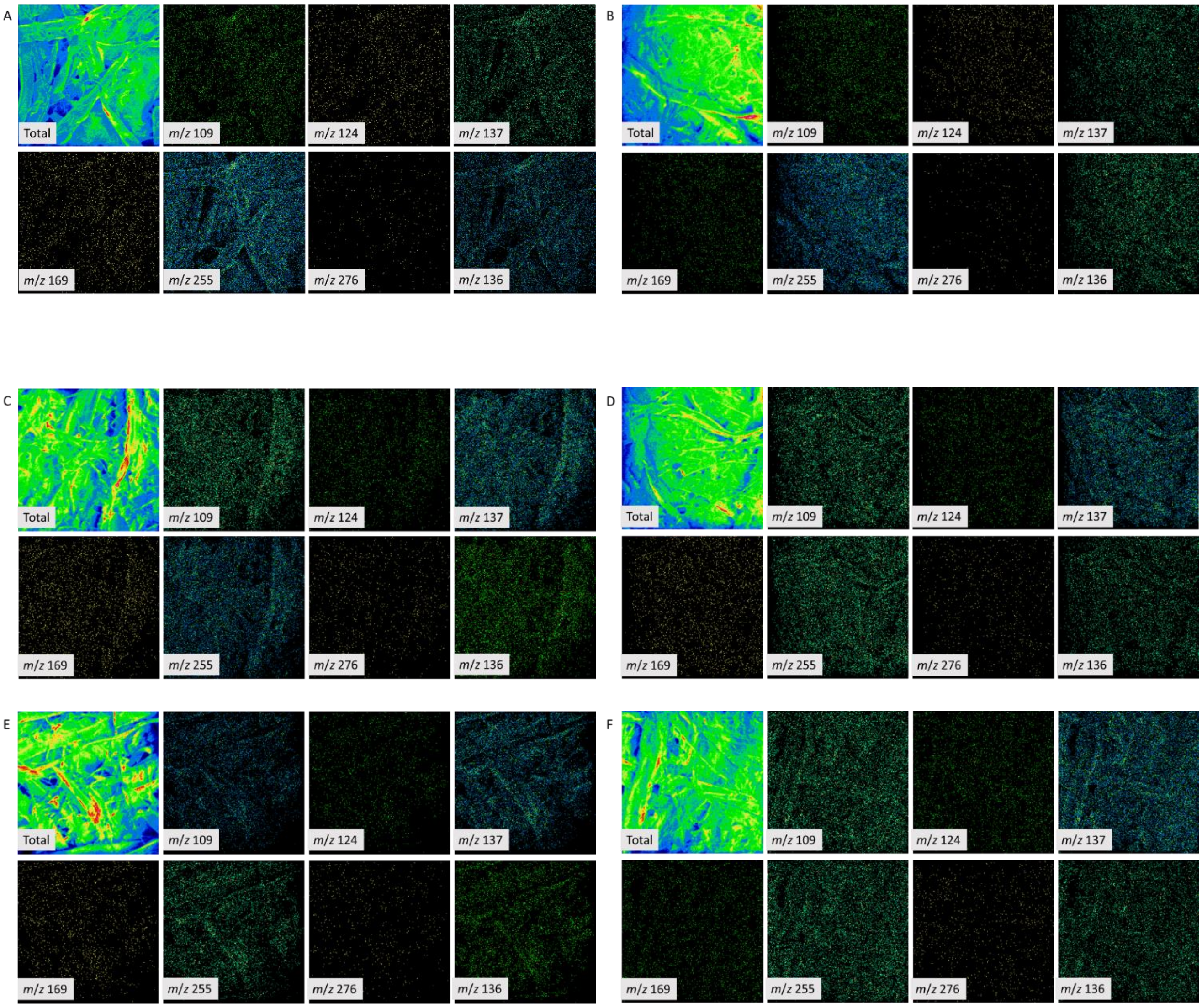

Figure S5. Negative ToF-SIMS images of the non-treated and tannin-rich bark extract (TW8.1) treated paper samples of 60 g/m^2 (A, C, E) and 130 g/m^2 (B, D, F). The peaks generated from TW8.1 acid (*m/z* of 124), and surface of paper handsheets (*m/z* of 109, 136, and 255). Note, that the negative candidate peak from tannic acid (not present in the sample) that was also *m/z* of 124. Total secondary ions (upper left corner in each sub-figure) represent surface structures of fiber matrices.

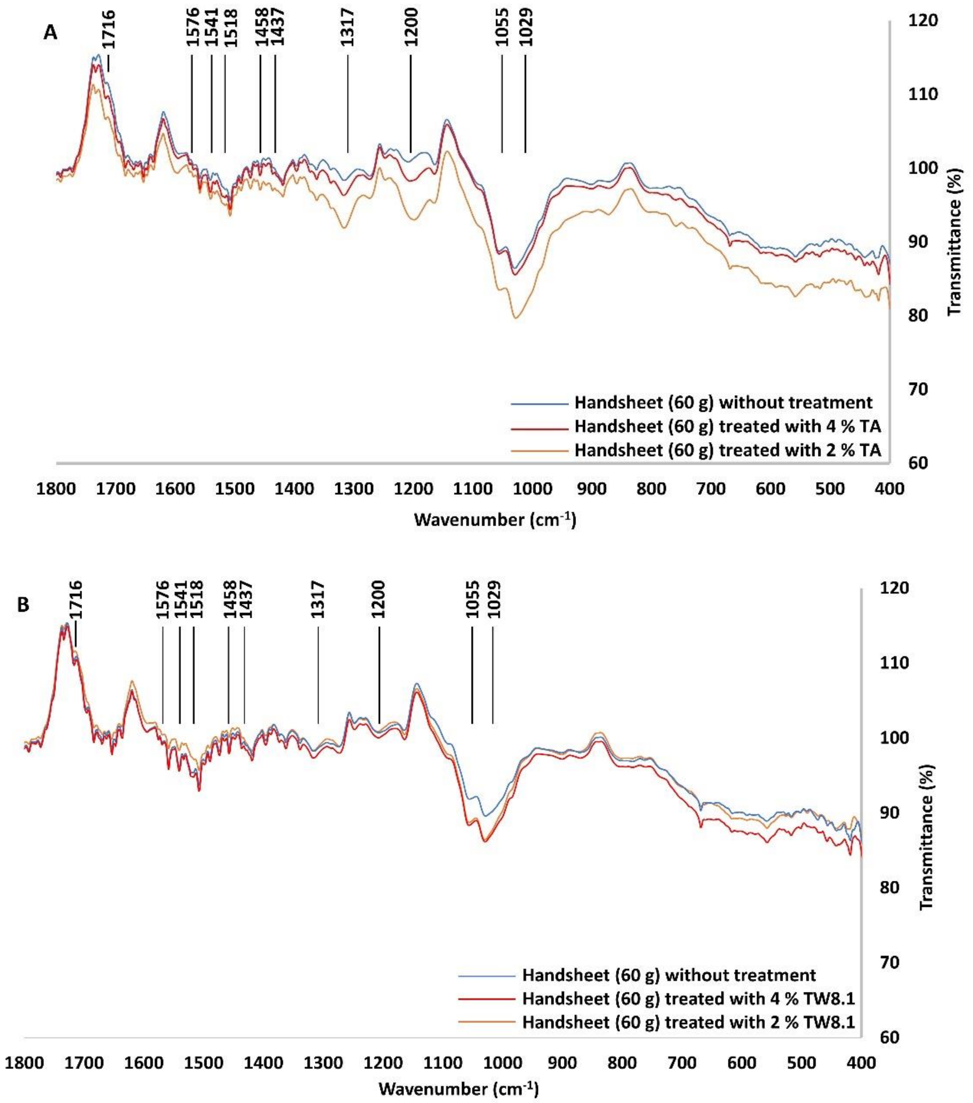

Figure S6. FTIR spectra of handsheet (60 g/m^2) treated with **(A)** 2% and 4% TA and **(B)** 2% and 4% TW.

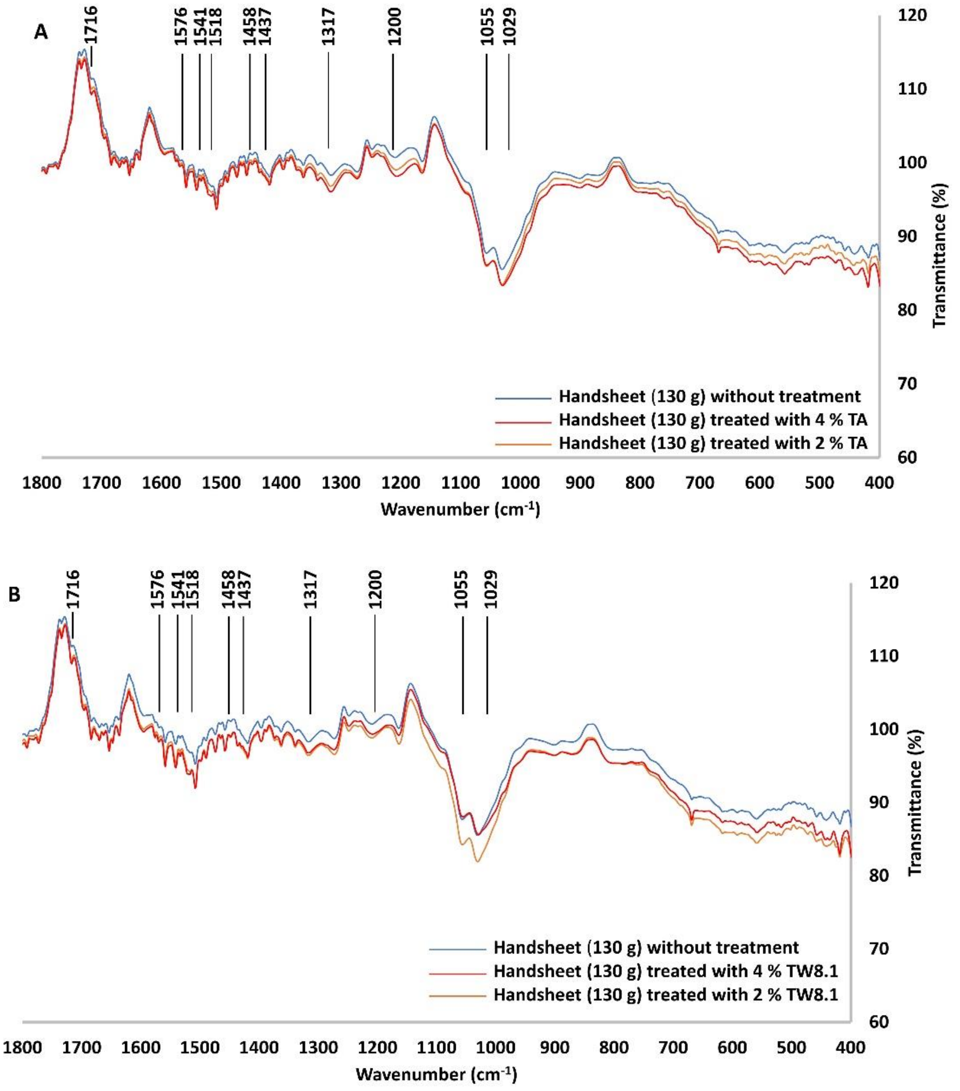

Figure S7. FTIR spectra of handsheet (130 g) treated with **(A)** 2% and 4% TA and **(B)** 2% and 4% TW.
